# Relative effects of melatonin and hydrogen sulfide treatments in mitigating salt damage in wheat

**DOI:** 10.3389/fpls.2024.1406092

**Published:** 2024-07-25

**Authors:** Sheen Khan, Ameena Fatima Alvi, Mehar Fatma, Abdulrahman Al-Hashimi, Adriano Sofo, Nafees A. Khan

**Affiliations:** ^1^ Plant Physiology and Biochemistry Laboratory, Department of Botany, Aligarh Muslim University, Aligarh, India; ^2^ Department of Botany and Microbiology, College of Science, King Saud University, Riyadh, Saudi Arabia; ^3^ Department of European and Mediterranean Cultures, Architecture, Environment, Cultural Heritage (DiCEM), University of Basilicata, Matera, Italy

**Keywords:** salinity, melatonin, hydrogen sulfide, antioxidants, carbohydrate metabolism, yield, wheat

## Abstract

Soil salinity poses a significant threat to agricultural productivity, impacting the growth and yield of wheat (*Triticum aestivum* L.) plants. This study investigates the potential of melatonin (MT; 100 µM) and hydrogen sulfide (H_2_S; 200 µM sodium hydrosulfide, NaHS) to confer the tolerance of wheat plants to 100 mM NaCl. Salinity stress induced the outburst of reactive oxygen species (ROS) resulting in damage to the chloroplast structure, growth, photosynthesis, and yield. Application of either MT or NaHS augmented the activity of antioxidant enzymes, superoxide dismutase, ascorbate peroxidase, glutathione reductase, and reduced glutathione (GSH) levels, upregulated the expression of Na^+^ transport genes (*SOS1, SOS2, SOS3, NHX1*), resulting in mitigation of salinity stress. Thus, improved stomatal behavior, gas-exchange parameters, and maintenance of chloroplast structure resulted in enhanced activity of the Calvin cycle enzymes and overall enhancement of growth, photosynthetic, and yield performance of plants under salinity stress. The use of DL-propargylglycine (PAG, an inhibitor of hydrogen sulfide biosynthesis) and *p*-chlorophenyl alanine (*p*-CPA, an inhibitor of melatonin biosynthesis) to plants under salt stress showed the comparative necessity of MT and H_2_S in mitigation of salinity stress. In the presence of PAG, more pronounced detrimental effects were observed than in the presence of *p*-CPA, emphasizing that MT was involved in mitigating salinity through various potential pathways, one of which was through H_2_S.

## 1 Introduction

The pronounced gradual increase in soil salinity is posing a severe threat to soil fertility, especially in regions characterized by arid climates, semiarid conditions, and extensive irrigation with saline water. By the middle of the twenty-first century, 50% of arable land is predicted to have been lost due to rising salinization (Mushtaq et al., 2020). Salinity is regarded as one of the most harmful abiotic stress factors affecting the growth and productivity of the majority of plants (Parihar et al., 2015). Salt buildup presents a challenging environment for plants, making it more difficult for them to take up water and nutrients from the soil and causing zones with low water potential to emerge. Long-term salt deposition causes physiological drought, making it difficult for plants to take up the water that is present in the soil (Porcel et al., 2016). Several signaling pathways, cellular processes including photosynthesis, and the activity of enzymes can be adversely affected by toxic ions accumulated in plants (Parihar et al., 2015). The anticipated consequences of global warming resulting from anthropogenic emissions of gases, such as CO_2_, are expected to worsen issues related to salt stress and desertification (Mushtaq et al., 2020). Elevated CO_2_ concentrations can generate surplus electrons that may react with O_2_, forming hazardous reactive oxygen species (ROS) (Mushtaq et al., 2020). As these ROS accumulate, they initiate the breakdown of lipids, leading to compromised membrane permeability (Mushtaq et al., 2020). This threatens chloroplast structures, the *psbA* and *psbB*-encoded proteins of photosystem (PS)II, and various enzymes crucial to plant metabolic pathways. This impact hinders photosynthesis and overall plant growth (Varghese et al., 2019; Sehar et al., 2021). Plants employ diverse adaptive processes to address various stressors, including ion exclusion and compartmentalization, synthesis of osmolytes, and increased antioxidant enzymes and phytohormone levels (Rasheed et al., 2021). One strategy to alleviate the adverse effects of salt stress on plants involves the exogenous supplementation of MT and H_2_S (Varghese et al., 2019; Kaya et al., 2023).

Melatonin, chemically known as N-acetyl-5-methoxytryptamine, is a tryptophan-derived pleiotropic molecule and a natural antioxidant present in all living organisms, spanning animals, plants and microbes (Guo et al., 2023; Arnao and Hernández-Ruiz, 2019). Due to its small amphiphilic nature, MT can easily traverse cell membranes, accessing both the cell nucleus and mitochondria (Arnao and Hernández-Ruiz, 2019). Functioning as a growth regulator and antioxidant, plant MT plays a significant role in the regulation of seed germination, shoot and root development, circadian rhythm, photosynthesis, and mitigation of both biotic and abiotic stress. The alleviating effects of MT on plant abiotic stress are often linked to the increased activity of antioxidant enzymes (Wang et al., 2018). For instance, Guo et al. (2023) demonstrated that MT mitigated salinity stress in alfalfa by enhancing antioxidant defense mechanisms and osmoregulation. In wheat plants subjected to salt stress, MT improved antioxidant activity and enhanced photosynthetic nitrogen use efficiency (p-NUE) (Khan et al., 2022b). Additionally, MT has been found to upregulate the expression of genes connected with photosynthesis, fatty acid biosynthesis, and carbohydrate metabolism in soybean (Wei et al., 2018). Under abiotic stress, MT improved stomatal characteristics (length, width, and area) and safeguarded the chloroplast structure, grana lamellae, and anatomical features of plants (Cui et al., 2017; Mohamed et al., 2020; Ahmad et al., 2021).

In recent years, there has been a paradigm shift in our understanding of H_2_S in plant biology. Contrary to previous beliefs that H_2_S was merely a harmful by-product of cellular metabolism, it is now recognized as a crucial gaseous signal molecule integral to various metabolic activities in plants (Kaya et al., 2023). Compelling evidence has emerged, highlighting the pivotal role of H_2_S in facilitating plant stress adaptation. It acts as a signal transducer, regulating oxidative stress and effectively modulating signaling pathways to promote stress resilience (Vojtovič et al., 2021). For example, H_2_S, through the modulation of the AsA-GSH system, alleviates salinity stress in wheat (Kaya et al., 2023). H_2_S is easily transported across cells and plays a crucial function in maintaining homeostasis in antioxidant system pools by supplying sulfur to cells or adjusting osmolyte concentrations (Pandey and Gautam, 2020). The complex interactions between H_2_S and plant hormones and signaling molecules—antagonistic or synergistic—are vital to stress-regulating mechanisms and significantly impact plant development dynamics (Luo et al., 2020). For example, H_2_S functions in conjunction with ethylene and nitric oxide signaling pathways to actively regulate redox homeostasis and protect photosynthesis during heat stress in rice (Gautam et al., 2022). H_2_S improves photosynthesis, photosynthetic enzyme gene expression, and thiol redox modification in *Spinacia oleracea* seedlings via stimulating chloroplast biogenesis (Chen et al., 2011). Through the persulfidation of PMA1(Plasma membrane H^+^-ATPase) in Arabidopsis (Ma et al., 2023), the maintenance of ion balance, and the transcriptional or posttranslational alteration of ROS-processing systems (Corpas and Palma, 2023)., H_2_S enhances salt tolerance.

MT interacts with diverse signaling molecules to safeguard plants from abiotic stressors (Samal et al., 2024; Khan et al., 2022a). Despite this, the precise mechanisms governing MT interaction with H_2_S during stress tolerance remain elusive. However, studies have begun to unravel this interaction, revealing MT’s role in modulating heat shock proteins and mitochondrial ATP synthase to enhance drought tolerance in an H_2_S-dependent manner (Khan et al., 2024). Additionally, the interplay between MT and H_2_S significantly influences carbohydrate metabolism, impacting the photosynthetic efficiency of wheat plants under heat stress (Iqbal et al., 2021). Further research has highlighted the collaborative function of NO and MT in regulating H_2_S-mediated tolerance to iron deficiency and salinity stress (Kaya et al., 2020), emphasizing the intricate network of interactions among MT, H_2_S, and other signaling molecules in shaping plant responses to abiotic stressors.

Wheat (*Triticum aestivum* L.) holds a crucial position as a primary cereal staple, supplying approximately 20% of the protein and 55% of its carbohydrate requirements for nearly 4.5 billion people worldwide (EL Sabagh et al., 2021). Predictions from different climate models indicate a potential 6% reduction in wheat production due to challenging environmental conditions (Asseng et al., 2015). The actual wheat yield per hectare often falls significantly, primarily attributed to factors like salinity (Pramila et al., 2019; EL Sabagh et al., 2021). Thus, enhancing the salt tolerance of wheat stands as a critical challenge in contemporary agriculture.

While previous research has investigated the stress-alleviating properties of MT and H_2_S individually, there is limited research available on the comparative potential of these two important signaling molecules in mitigating stress. This study compared the efficacy of MT and H_2_S and their relationship in alleviating salinity stress in wheat. Our investigation encompasses an in-depth analysis of plant growth, photosynthetic capacity, antioxidant mechanisms, carbohydrate metabolism, ion-homeostasis, leaf ultrastructure, and yield under salinity conditions.

## 2 Material and methods

### 2.1 Plant growth conditions and treatments

Wheat seeds (*Triticum aestivum* L. cv. GOAL) were planted in earthen pots with a diameter of 23 cm, filled with acid-washed sand post-sterilization using a diluted hypochlorite solution. The cultivar selected for this experiment was chosen based on our prior research, which involved screening for salt tolerance by assessing changes in photosynthesis, growth, and oxidative parameters (Khan et al., 2022b). The pots were placed in the experimental area of the Department of Botany at Aligarh Muslim University, Aligarh (India). The growth conditions included natural day/night cycles with photosynthetically active radiations (PAR) at an average of 680 μmol m^-2^ s^-1^, day/night temperatures averaging 20°C/17°C (± 3°C), and a relative humidity of 65 ± 5%. Each pot accommodated four plants and received 300 ml of Hoagland nutrient solution every alternate day. Stress was induced by adding 100 mM NaCl at 10 days after sowing (DAS) and applied three times on alternate days over a week. A solution of 100 μM MT and 200 µM NaHS (H_2_S donor) was individually sprayed on both the unstressed and stressed plants. Additionally, inhibitors, 1 mM PAG (DL-propargylglycine - H_2_S synthesis inhibitor) and 10 μM *p*-CPA (p-chlorophenyl alanine - melatonin synthesis inhibitor) were sprayed to stressed plants treated with MT and H_2_S, respectively, at 15 DAS and continued on alternate days for one week. The concentrations of MT and NaHS were based on our previous studies (Khan et al., 2022b; Gautam et al., 2022), while PAG and *p*-CPA concentrations were derived from Sun et al. (2021). Teepol (0.5%) surfactant was included in the control and treatment solutions. The experimental design featured eight treatments: (1) control, (2) NaCl, (3) MT, (4) NaHS, (5) MT +NaCl, (6) NaHS +NaCl, (7) MT +NaCl +PAG, and (8) NaHS +NaCl +*p*-CPA. The observations on morphophysiological, biochemical, and molecular aspects were recorded at 30 DAS, while yield traits were assessed at the time of harvesting. The treatments were organized in a completely randomized block design (CRBD), with four replicates for each treatment (n = 4).

### 2.2 Measurement of growth attributes

Leaf area measurements were conducted using a leaf area meter (LA 211, Systronics, New Delhi, India). To determine plant dry weight, the plants were cleaned and subjected to drying in an oven at 80°C until a constant weight was achieved.

### 2.3 Determination of oxidative biomarkers

The quantification of leaf hydrogen peroxide (H_2_O_2_) content was carried out using the approach outlined by Okuda et al. (1991). The assessment of lipid peroxidation was performed by determining the levels of thiobarbituric acid reactive substances (TBARS), following the procedure described by Dhindsa et al. (1981). Additional information on the methodology can be found in **Supplementary Material S1**.

### 2.4 Determination of the activity of antioxidant enzyme and GSH content

Fresh leaves were homogenized in a chilled mortar and pestle using an extraction buffer composed of 0.05% (v/v) Triton X-100 and 1% (w/v) PVP in a potassium phosphate buffer (100 mM, pH 7.0). The resulting supernatant, obtained post-centrifugation, served as the substrate for the assays of SOD (EC; 1.15.1.1) and GR (EC; 1.6.4.2) enzymes. In the case of the APX (EC; 1.11.1.11) assay, 2.0 mM ascorbate was added to the extraction buffer.

The activity of SOD was assessed according to the procedures outlined by Beyer and Fridovich (1987) and Giannopolitis and Ries (1977). APX activity was determined by monitoring the reduction in ascorbate absorbance at 290 nm, following the method of Nakano and Asada (1981). The GR activity was measured by observing the GSH-dependent oxidation of NADPH at 340 nm, using the method described by Foyer and Halliwell (1976). Comprehensive details of these methods are available in **Supplementary File 1**.

GSH content was determined using a modified version of Anderson’s method (1985). Further details of this procedure are provided in **Supplementary Material S1**.

### 2.5 Content of sodium and potassium ions

Leaf samples weighing 500 mg were subjected to digestion using 19 ml of a Tri acid mixture. This mixture consisted of 18 M H_2_SO_4_, 16 M HNO_3_, and 11.65 M HClO_4_ in a proportion of 5:10:4. The resulting digested samples were utilized for assessing the content of Na^+^ and K^+^ in the leaves through the application of a flame photometer (Khera 391, Khera instruments).

### 2.6 Measurement of relative water content

RWC of the leaf was determined following the methodology outlined by Barrs and Weatherley (1962). Fresh leaves were promptly collected and weighed using a standardized balance. Subsequently, these leaves were immersed in water in separate Petri dishes for a duration of 12 hours. The turgid weight was computed by weighing the leaves while they were still wet. The leaf samples were then subjected to oven-drying at 80°C for 48 hours, and the resulting dry weight was recorded. The formula used for calculating RWC:


RWC (%)=(Fresh weight−Dry weight)/(Turgid weight−Dry weight)×100

### 2.7 Measurement of root activity

Root activity was assessed using the triphenyl tetrazolium chloride (TTC) technique detailed by Zhang et al. (2013). Roots with tips weighing 0.5 g were submerged in a solution comprising 10 ml of 0.4% (w/v) TTC and 10 ml sodium phosphate buffer (pH 7.0). This immersion occurred in the dark for over 4 hours at 37°C until the apical section exhibited complete whitening. Subsequently, the root samples were extracted with 5 ml of ethyl acetate. The resulting solution was transferred and adjusted to a volume of 10 ml by adding ethyl acetate. Root activity was quantified based on the intensity of TTC reduction; the extracted solution from the roots was then assessed at 485 nm. The formula for root activity calculation is given:


Root activity=amount of TTC reduction(µg)/fresh root weight(g)×time(h)

### 2.8 Determination of melatonin and hydrogen sulfide content

The method for melatonin content was adopted from Sehar et al. (2023), and the method for hydrogen sulfide from Xie et al. (2014). Details of the methodology are given in **Supplementary Material S1**.

### 2.9 Scanning electron microscopy

Healthy and fully developed leaf samples underwent fixation for over 4 h in a solution comprising 2.5% glutaraldehyde and 2% paraformaldehyde in 0.1 M phosphate buffer (pH 7.0). Subsequently, the samples were rinsed three times for 15 minutes each. Post-fixation involved a 1-h phosphate buffer (pH 7.0) treatment containing 1% osmium tetraoxide, followed by another three 15-minute rinses in the same phosphate buffer. To achieve complete dehydration, the samples were sequentially immersed in an ethanol series of 50, 70, 80, 90, 95, and 100% for 15-20 minutes each. The samples were then transferred to pure iso-amyl acetate and left for 1 h. Finally, after dehydration, the samples were mounted in the Carl Zeiss EVO 40 scanning electron microscope (Germany) under extra high tension or high voltage at 15 kV and a magnification of 1000×. Stomatal aperture dimensions, including length and width, were measured using a micrometer scale.

### 2.10 Transmission electron microscopy

Transmission electron microscopy (TEM) was employed to elucidate the chloroplast’s ultrastructure. Leaf segments measuring 1-2 mm were initially subjected to primary fixation in a 2.5% glutaraldehyde solution in 50 mM phosphate buffer (pH 6.8). This was followed by treatment with 1% osmium tetroxide for 30 minutes in 50 mM sodium cacodylate buffer (pH 7.2) and subsequent dehydration using a graded series of ethanol (30–100%, v/v). After the ethanol dehydration steps, the tissue underwent replacement with propylene oxide and embedded in Spurr resin. Ultrathin slices were then prepared using the Leica EM UC6 ultramicrotome (Leica, Wetzlar, Germany). Staining was accomplished using uranyl acetate and lead citrate before examination with a JEM-2100F field emission electron microscope (Jeol Ltd.; Tokyo, Japan) at 120 kV.

### 2.11 Determination of total soluble sugars, total non-structural carbohydrates, and starch content

The estimation of total soluble sugars followed the procedure outlined by Xu et al. (2015) while the measurement of starch content was conducted using the methodology of Kuai et al. (2014). Comprehensive details of these methods are provided in **Supplementary File 1**. The non-structural carbohydrate (NSC) content was determined by the addition of both soluble sugar and starch content.

### 2.12 Assay of activity of the Calvin cycle enzymes

The activity of Rubisco, FBPase, and SBPase was assessed using the method outlined by Usuda (1985), Zhang et al. (2014), and Harrison et al. (1997), respectively. Details of the protocol are given in **Supplementary Material S1**.

### 2.13 Measurement of photosynthetic characteristics, chlorophyll fluorescence, and yield attributes

Net photosynthesis, stomatal conductance, and intercellular CO_2_ concentration were measured in fully expanded leaves utilizing an Infrared Gas Analyzer (CID-340, Photosynthesis System, Bioscience). Measurements were conducted between 11 a.m. and 12 p.m. under light-saturating conditions (PAR) of 760 μmol m^2^ s^-1^ at a temperature of 22°C and with a relative humidity of approximately 70%. Chlorophyll content was determined using a SPAD chlorophyll meter (502 DL PLUS, Spectrum Technologies).

The maximal quantum yield of PSII efficiency (Fv/Fm) was assessed using a chlorophyll fluorometer (Junior-PAM, Heinz Walz, GmbH, Effeltrich, Germany) on fully expanded leaves. After 30 minutes of dark adaptation, maximum fluorescence (Fm) and variable fluorescence (Fv) were determined. Fv was calculated as (Fm–Fo), with Fo measured under weak modulated light (PPFD of 0.1 µmol photons m^−2^ s^−1^). Fm was obtained using a saturating pulse (>6000 µmol photons m^−2^ s^−1^). The maximal quantum yield of PSII efficiency was then calculated as Fv/Fm = (Fm–Fo)/Fm.

Manual recordings were made for spike length, the number of spikelets per spike, total grain per spike, and the weight of 1000 grains at crop maturity.

### 2.14 RNA extraction and quantitative RT-PCR

Total RNA was extracted from leaves using TRIzol reagent from Ambion, Life Technologies, following the manufacturer’s protocol. RNA quantity was assessed using a Nanodrop spectrophotometer from Thermo Scientific, Waltham, MA, USA. Subsequently, first-strand cDNA was synthesized from 1 μg of total RNA from both control and treated samples, and the template was synthesized using a reaction mixture containing 20 U/μL Moloney Murine Leukemia virus reverse transcriptase (MuMLV) enzyme from Fermentas, Maryland, USA, at 42°C for 50 min, followed by 70°C for 10 min. The reverse transcription process was performed using 2.5 μM Oligo (dT) 18 primer (Fermentas, USA) and 10 mM dNTPs. Primers for gene expression analysis were designed using IDT online primer design software, and the cDNA sequences of target genes were sourced from NCBI.

Real-time PCR (RT-PCR) was conducted on a thermal cycler (Light cycler 480 II, Roche, Germany) using a 96-well reaction plate (Roche, Mannheim, Germany). The setup consisted of reaction a mixture (20 µL) of ×10 reaction buffer, 10 µL cDNA template, 1 mM MgCl_2_, 2 mM dNTPs, 1 µL Sybr green (×10) (Thermo Fisher Scientific, Waltham, MA, USA) 0.35 µM each of forward and reverse primers and 5 U Taq polymerase (Bio-rad, Hercules, CA, USA). β-actin forward and β-actin reverse primers were employed to amplify an actin DNA fragment, serving as an internal control for normalization across all genes. PCR cycling conditions included denaturation at 95°C for 3 minutes, 40 cycles of 95°C for 20 seconds, 66°C for 1 minute, and 72°C for 1 minute, and a final extension step at 72°C for 5 minutes. Amplified products were analyzed on a 1.2% agarose gel, and the specificity of the amplicons was confirmed through melting curve analysis (60 to 95°C) after 40 cycles. Data analysis involved a comparison of gene expression levels in treated samples to those in untreated controls, normalized against the internal control (actin). The primer pairs utilized for quantitative RT-PCR are given in **Table S1** of the **Supplementary Material**. The relative amount of the target gene expression was determined by the 2^−ΔΔCT^ method.

### 2.15 Statistical analysis

Statistical analysis of the data obtained from the experiments employing a completely randomized block design was performed using analysis of variance (ANOVA) through SPSS 17.0 for Windows. The results are presented as mean ± standard error (n = 4). The least significant difference (LSD) was computed at *p*< 0.05 for significant data. Bars sharing the same letter were not significantly different based on the LSD test at *p*< 0.05. Principal Component Analysis (PCA) was executed using OriginPro software. To illustrate the most significant variance in the datasets, biplots were created by considering the first two components (PC1 and PC2).

## 3 Results

### 3.1 Plant growth parameters

Plants subjected to salt stress exhibited a decrease in leaf area and plant dry weight by 47.2% and 48.2%, respectively, relative to the control (**Table 1**). The treatment of MT and NaHS alone under unstressed conditions improved growth traits compared to the control. Moreover, when MT and NaHS were applied individually to salt-stressed plants, there was a significant enhancement in growth attributes under stressed conditions, by increasing leaf area by 72.5 and 52.6% and plant dry weight by 73.2 and 53.3%, respectively, relative to plant’s subjected to salt stress alone. Externally applied MT showed a more prominent effect compared to NaHS. However, the potential influences of MT and NaHS on these parameters were markedly attenuated by the inhibitors, namely PAG and *p*-CPA. Notably, a more pronounced negative effect was observed under the treatment involving MT + NaCl + PAG, as opposed to the treatment comprising NaHS + NaCl + *p*-CPA.

**Table 1:** Leaf area, plant dry weight, chlorophyll content, net photosynthesis, stomatal conductance, intercellular CO_2_ concentration, and maximal quantum yield of PSII efficiency (Fv/Fm) of wheat (*Triticum aestivum* L. cv. GOAL) at 30 days after sowing (DAS). Plants were treated with 100 µM melatonin (MT) or 200 µM sodium hydrosulfide (NaHS; H_2_S donor) in the presence or absence of 100 mM NaCl or were treated with 1mM DL-propargylglycine (PAG; H_2_S synthesis inhibitor) with MT and NaCl or with 10 μM *p*-chlorophenylalanine (*p*-CPA, melatonin synthesis inhibitor) with NaHS and NaCl.

Treatments	Leaf area (cm^2^ plant^-1^)	Plant dry weight (g plant^-1^)	Chlorophyll content (SPAD Value)	Net photosynthesis (µmol CO_2_ m^-2^ s^-1^)	Stomatal conductance (mmol CO_2_ m^-2^ s^-1^)	Intercellular CO_2_ concentration (µmol CO_2_ mol^-1^)	Maximum quantum efficiency of PSII
**Control**	32.4 ± 1.03b	0.87 ± 0.04c	39.2 ± 1.32b	14.3 ± 1.03b	402 ± 15.1b	308 ± 13.2bc	0.78 ± 0.03b
**NaCl**	17.1 ± 0.45g	0.45 ± 0.03h	23.5 ± 1.15f	7.2 ± 0.45e	245 ± 12.6g	191 ± 12.3g	0.43 ± 0.03g
**MT**	39.2 ± 1.12a	1.24 ± 0.06a	43.6 ± 1.36a	17.1 ± 1.12a	435 ± 15.7a	337 ± 14.1a	0.91 ± 0.04a
**NaHS**	38.5 ± 1.15a	1.09 ± 0.06b	42.7 ± 1.36a	16.2 ± 1.15a	411 ± 15.2ab	325 ± 13.5ab	0.82 ± 0.04b
**MT + NaCl**	29.5 ± 0.53c	0.78 ± 0.04d	34.5 ± 1.27c	12.6 ± 0.53c	373 ± 14.4c	297 ± 12.8c	0.71 ± 0.03c
**NaHS + NaCl**	26.1 ± 0.68d	0.69 ± 0.03e	30.3 ± 1.25d	11.1 ± 0.68d	326 ± 14.1d	274 ± 13.1d	0.64 ± 0.04d
**MT +NaCl + PAG**	20.3 ± 0.51f	0.54 ± 0.02fg	26.3 ± 1.11e	8.6 ± 0.39ef	270 ± 13.3f	214 ± 12.4f	0.51 ± 0.02f
**NaHS +NaCl + *p*-CPA**	22.1 ± 0.54e	0.60 ± 0.02f	28.2 ± 1.15de	9.5 ± 0.42f	296 ± 13.5e	241 ± 12.6e	0.58 ± 0.02e

The foliar treatments of MT, NaHS, PAG, and *p*-CPA were given at 15 DAS. The data is displayed as the mean of treatments ± standard error (n = 4). Values with identical letters showed no significant differences according to the LSD test at a significance level of *p*< 0.05.

### 3.2 Photosynthesis-related parameters

Salt stress led to a reduction in chlorophyll content, net photosynthesis, stomatal conductance, intercellular CO_2_ concentration, and maximum quantum yield efficiency of PSII by 40.1%, 49.7%, 39.1%, 37.9%, 44.8%, respectively, in comparison to the control (**Table 1**). The treatment of MT and NaHS alone under unstressed conditions improved photosynthetic traits compared to the control. Moreover, when MT and NaHS were applied individually to salt-stressed plants, there was a significant enhancement in chlorophyll content by (46.8 and 28.9%), net photosynthesis (75.0 and 54.1%), stomatal conductance (52.2 and 33.1%), intercellular CO_2_ concentration (55.4 and 43.5%), maximum PSII efficiency (65.1 and 48.8%) respectively, in comparison to plants treated with salt alone. Externally applied MT significantly mitigated the adverse impacts of salinity stress on photosynthetic characteristics to a greater extent compared to NaHS. However, the potential influences of MT and NaHS on these parameters were markedly attenuated by the inhibitors, namely PAG and *p*-CPA. Notably, a more pronounced negative effect was observed under the treatment involving MT + NaCl + PAG, as opposed to the treatment comprising NaHS + NaCl + *p*-CPA. This suggests that H_2_S functions as one of the signaling molecules in the MT-mediated response.

### 3.3 Sodium, potassium ion, and relative water content

**Figure 1:**
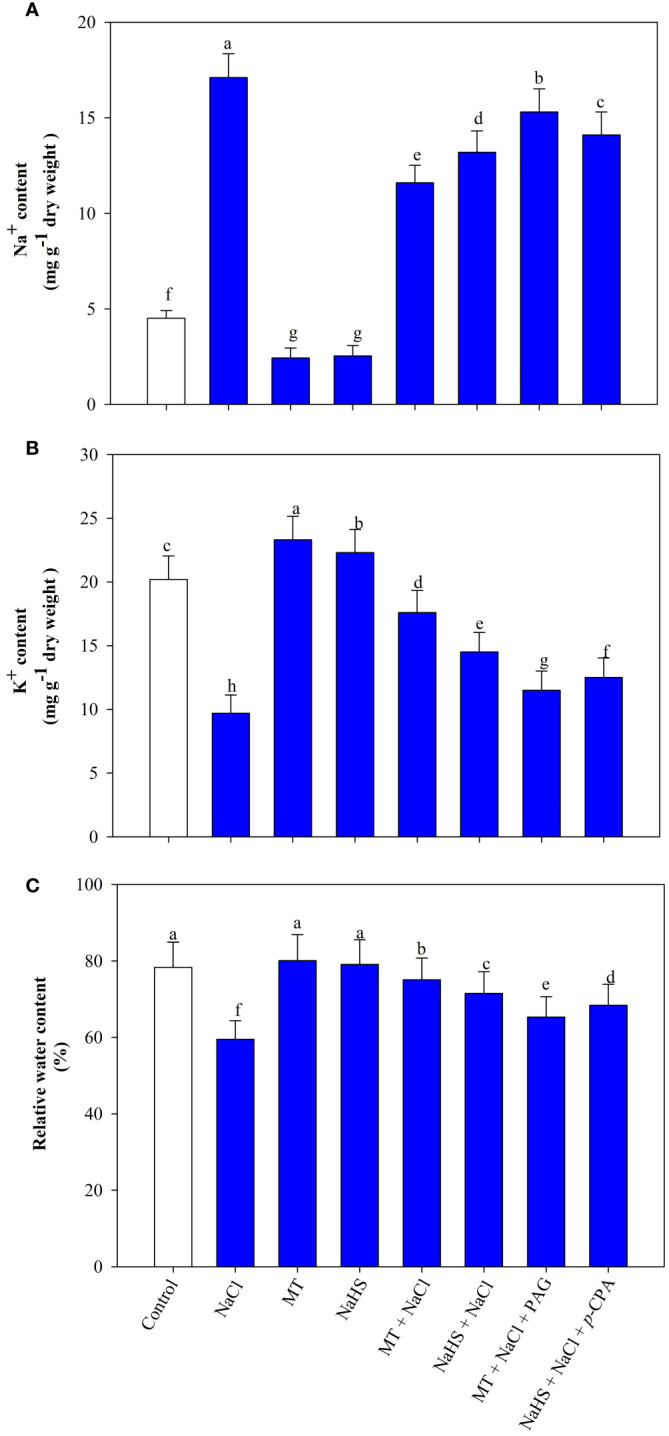
Sodium (Na^+^) content **(A)**, potassium (K^+^) content **(B)**, relative water content **(C)** of wheat (*Triticum aestivum* L. cv. GOAL) at 30 days after sowing (DAS). Plants were treated with 100 µM melatonin (MT) or 200 µM sodium hydrosulfide (NaHS; H_2_S donor) in the presence or absence of 100 mM NaCl or were treated with 1mM DL-propargylglycine (PAG; H_2_S synthesis inhibitor) with MT and NaCl or with 10 μM *p*-chlorophenylalanine (*p*-CPA, melatonin synthesis inhibitor) with NaHS and NaCl. The foliar treatments of MT, NaHS, PAG, and *p*-CPA were given at 15 DAS. The data is displayed as the mean of treatments ± standard error (n = 4). Values with identical letters showed no significant differences according to the LSD test at a significance level of *p*< 0.05.

Salt stress induced a substantial decrease in leaf K^+^ content and an elevation in Na^+^ ion levels. Individual supplementation of MT and NaHS, under stress conditions, enhanced K^+^ content by 81.4% and 49.5%, respectively, and reduced Na^+^ content by 32.2% and 22.8%, respectively, in comparison to plants exposed solely to stress. Upon employing inhibitors in salt-exposed plants, the concurrent application of MT and PAG, as well as NaHS and p-CPA, results in decreased K^+^ content and increased Na^+^ ion content compared to plants exposed to MT +NaCl and NaHS +NaCl treatment, respectively. Notably, the combined application of H_2_S inhibitor with MT under salt-stress demonstrated maximum reduction in K^+^ content and increment in Na^+^ content (**Figures 1A, B**).

Salinity caused a 24.1% reduction in leaf RWC. Application of MT or NaHS improved RWC, with MT showing a remarkable 26.2% recovery and NaHS a notable 20.1% improvement in salt-stressed plants. However, in salt-exposed plants, the application of MT+PAG and NaHS +*p*-CPA applications resulted in reduced RWC, with MT +NaCl +PAG resulting in a more pronounced reduction (**Figure 1C**).

### 3.4 Root activity

**Figure 2:**
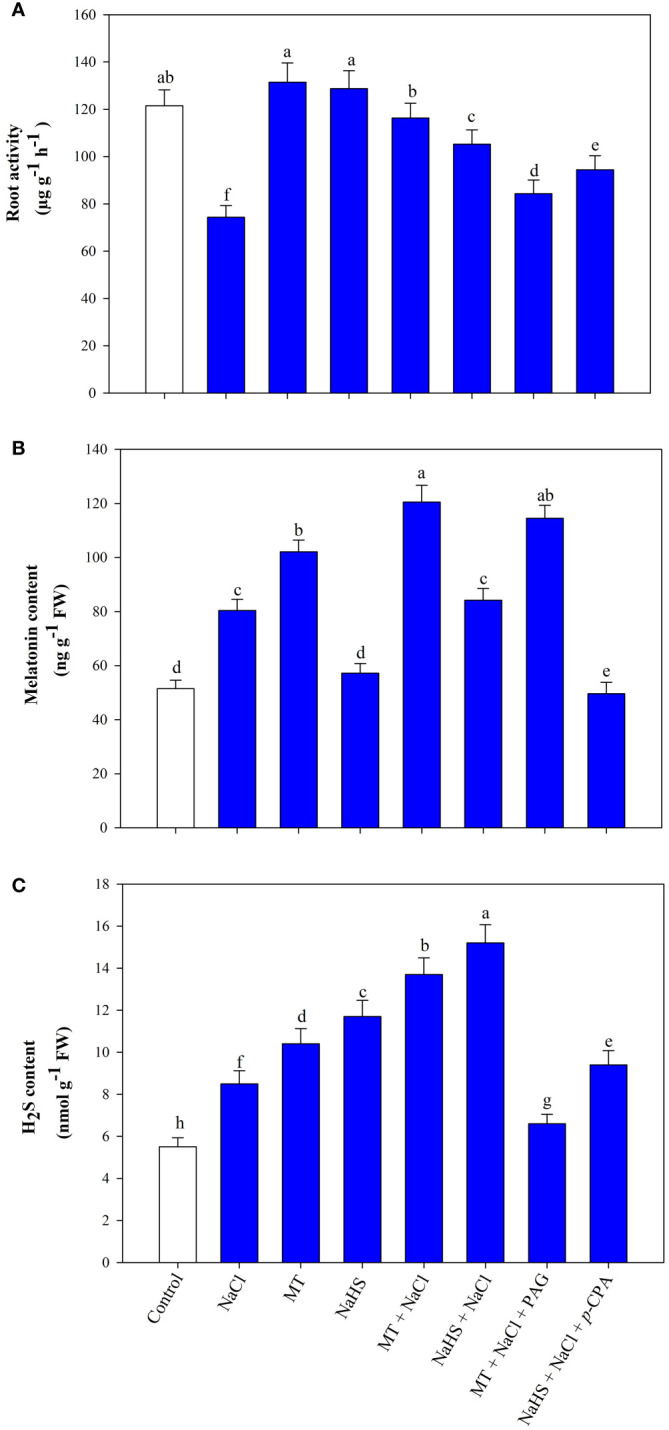
Root activity **(A)**, melatonin content **(B)**, hydrogen sulfide (H_2_S) content **(C)** of wheat (*Triticum aestivum* L. cv. GOAL) at 30 days after sowing (DAS). Plants were treated with 100 µM melatonin (MT) or 200 µM sodium hydrosulfide (NaHS; H_2_S donor) in the presence or absence of 100 mM NaCl or were treated with 1mM DL-propargylglycine (PAG; H_2_S synthesis inhibitor) with MT and NaCl or with 10 μM *p*-chlorophenylalanine (*p*-CPA, melatonin synthesis inhibitor) with NaHS and NaCl. The foliar treatments of MT, NaHS, PAG, and *p*-CPA were given at 15 DAS. The data is displayed as the mean of treatments ± standard error (n = 4). Values with identical letters showed no significant differences according to the LSD test at a significance level of *p*< 0.05.

Root activity, encompassing absorption, synthesis, oxidation, and reduction capacities, serves as a vital physiological indicator reflecting the dynamic functions of the root system. Salt stress led to a significant 38.8% reduction in root activity compared to the control (**Figure 2A**). MT application during stress enhanced root activity by 56.5% and NaHS by 41.5% relative to plants exclusively subjected to NaCl treatment. Additionally, using inhibitors in combination with MT or NaHS under stress conditions reduces the root activity, with MT +NaCl +PAG treatment plants showing maximum reduction in contrast to NaHS +NaCl +*p*-CPA treatment.

### 3.5 Melatonin and H_2_S content

Salinity stress led to a 56.1% increase in the endogenous levels of MT and a 54.5% increase in H_2_S in wheat leaves compared to control plants (**Figures 2B, C**). Application of exogenous MT enhanced endogenous MT and H_2_S content in both stressed and unstressed conditions. However, treatment of NaHS does not show much impact on MT content but increases the endogenous H_2_S levels in leaves under control and salt-stressed conditions. In salt-stressed plants, MT and NaHS application resulted in a 49.9% and 4.72% increase in MT content and a 61.2% and 78.8% increase in H_2_S content, respectively, compared to plants treated with NaCl alone. In plants treated with the MT +NaCl +PAG, not much effect is seen in MT content compared to MT +NaCl, but H_2_S content was even lower than in plants treated with NaCl alone. Additionally, in plants treated with NaHS +NaCl +*p*-CPA, the MT content was lower than in plants subjected to NaCl stress, while the H_2_S level was higher than in plants experiencing only salt stress but lower than in NaHS +NaCl treated plants.

### 3.6 Oxidative stress and antioxidants

Oxidative stress levels were assessed through the measurement of H_2_O_2_ and TBARS. Under salt stress, plants exhibited a substantial increase in H_2_O_2_ by 171.7% and TBARS content by 160.0% compared to control plants (**Table 2**). However, applying either MT or NaHS effectively decreased H_2_O_2_ and TBARS levels in both stressed and unstressed conditions. MT and NaHS treatments demonstrated a mitigating effect on salt-induced oxidative stress, evident in the significant reductions of H_2_O_2_ (50.8% and 31.9%) and TBARS (43.3% and 30.7%) levels, respectively, compared to only salt-stressed plants. These outcomes indicate that individual treatments with MT and NaHS effectively alleviate salt-induced oxidative stress by reducing the accumulation of H_2_O_2_ and TBARS.

**Table 2:** Content of hydrogen peroxide (H_2_O_2_), thiobarbituric acid reactive substances (TBARS), reduced glutathione (GSH) and activity of superoxide dismutase (SOD), ascorbate peroxidase (APX), and glutathione reductase (GR) of wheat (*Triticum aestivum* L. cv. GOAL) at 30 days after sowing (DAS).

Treatments	H_2_O_2_	TBARS	GSH	SOD	APX	GR
	nmol g^-1^ FW			U mg^-1^ protein min^-1^		
**Control**	15.2 ± 0.70f	5.5 ± 0.23f	262 ± 15.7f	7.1 ± 0.42h	1.6 ± 0.21g	2.1 ± 0.12g
**NaCl**	41.3 ± 1.81a	14.3 ± 0.81a	310 ± 16.1e	12.2 ± 0.53e	3.1 ± 0.51e	3.1 ± 0.22e
**MT**	10.6 ± 0.52g	3.4 ± 0.12g	321 ± 19.5de	10.4 ± 0.41f	2.4 ± 0.34f	2.7 ± 0.15f
**NaHS**	12.1 ± 0.52fg	4.1 ± 0.15g	315 ± 19.2e	9.1 ± 0.63g	2.2 ± 0.32f	2.4 ± 0.15fg
**MT + NaCl**	20.3 ± 0.91e	8.1 ± 0.42e	467 ± 25.4a	17.8 ± 0.82a	7.1 ± 0.61a	5.5 ± 0.37a
**NaHS + NaCl**	28.1 ± 0.93d	9.9 ± 0.42d	412 ± 20.6b	16.5 ± 0.78b	6.2 ± 0.53b	4.5 ± 0.32b
**MT +NaCl + PAG**	33.5 ± 1.91b	12.5 ± 0.84b	351 ± 14.4cd	13.5 ± 0.33d	3.9 ± 0.11d	3.5 ± 0.11d
**NaHS+ NaCl + *p*-CPA**	31.2 ± 1.72c	11.2 ± 0.83c	378 ± 15.5c	15.5 ± 0.36c	5.1 ± 0.12c	4.1 ± 0.14c

Plants were treated with 100 µM melatonin (MT) or 200 µM sodium hydrosulfide (NaHS; H_2_S donor) in the presence or absence of 100 mM NaCl or were treated with 1mM DL-propargylglycine (PAG; H_2_S synthesis inhibitor) with MT and NaCl or with 10 μM *p*-chlorophenylalanine (*p*-CPA, melatonin synthesis inhibitor) with NaHS and NaCl. The foliar treatments of MT, NaHS, PAG, and *p*-CPA were given at 15 DAS. The data is displayed as the mean of treatments ± standard error (n = 4). Values with identical letters showed no significant differences according to the LSD test at a significance level of *p*< 0.05.

Examining enzymatic antioxidants, including SOD (superoxide dismutase), APX (ascorbate peroxidase), and GR (glutathione reductase), as well as the non-enzymatic antioxidant reduced GSH (glutathione) content, revealed that salt stress significantly increased the activity of SOD, APX, and GR by 71.8%, 93.7%, 47.6%, respectively and content of GSH by 18.3%, respectively, relative to control plants. Using either MT or NaHS further augmented enzyme activity and GSH content under both stressed and unstressed conditions. Under stress, MT and NaHS enhanced SOD activity by 45.9% and 35.2%, APX by 129.0% and 100.0%, GR by 77.4% and 45.1%, and GSH content by 50.6% and 32.9%, respectively, compared to plants treated only with salt (**Table 2**). The application of MT effectively alleviated the deleterious effects of salinity by enhancing antioxidant machinery and reducing oxidative stress to a greater degree than NaHS. Contrastingly, plants treated with MT + NaCl + PAG exhibited higher oxidative stress and reduced activity of antioxidants and content of non-enzymatic antioxidant than those treated with NaHS + NaCl + *p*-CPA. This observation implies that H_2_S is required for modulating oxidative stress and antioxidant defense mechanisms by MT under stress responses.

### 3.7 Relative expression of SOS-pathway and *NHX1* gene

**Figure 3:**
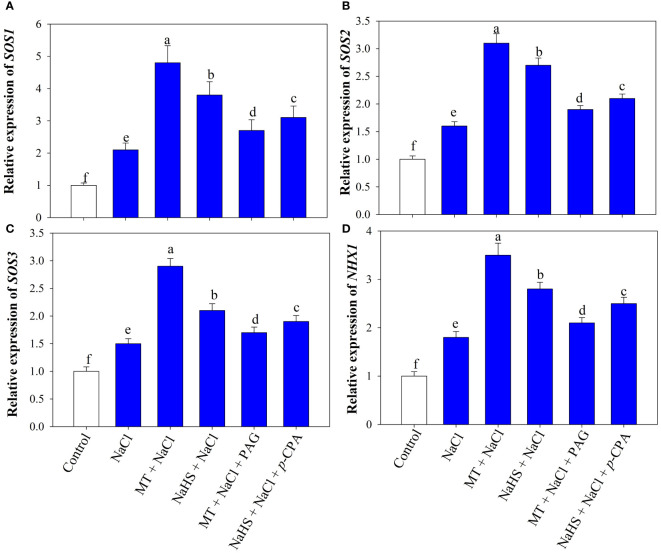
Relative expression of *SOS1* **(A)**, *SOS2* **(B)**, *SOS3* **(C)**, *NHX1* **(D)** of wheat (*Triticum aestivum* L. cv. GOAL) at 30 days after sowing (DAS). Plants were treated with 100 µM melatonin (MT) or 200 µM sodium hydrosulfide (NaHS; H_2_S donor) in the presence or absence of 100 mM NaCl or were treated with 1mM DL-propargylglycine (PAG; H_2_S synthesis inhibitor) with MT and NaCl or with 10 μM *p*-chlorophenylalanine (*p*-CPA, melatonin synthesis inhibitor) with NaHS and NaCl. The foliar treatments of MT, NaHS, PAG, and *p*-CPA were given at 15 DAS. The data is displayed as the mean of treatments ± standard error (n = 4). Values with identical letters showed no significant differences according to the LSD test at a significance level of *p*< 0.05.

Investigating the impact of MT and NaHS on the expression of Na^+^ transport genes, we assessed the relative expression of the SOS pathway and *NHX1* genes (**Figures 3A-D**). Under NaCl treatment, there was a substantial upregulation in the expression of *SOS1, SOS2, SOS3*, and *NHX1 by* 2.1, 1.6, 1.5, and 1.8fold, respectively, in comparison to control plants. When MT and NaHS were individually applied under salt stress there was a further notable enhancement in the expression of the SOS pathway and *NHX1* gene in wheat leaves, surpassing the levels observed in NaCl-treated plants alone. Notably, MT exhibited a more profound effect compared to NaHS. On the contrary, plants treated with MT +NaCl+ PAG displayed decreased transcription levels of these genes compared to those treated with NaHS +NaCl +*p*-CPA. This highlights the differential impact of MT and NaHS, with MT demonstrating a more substantial influence through H_2_S on the expression of the examined genes under salt stress.

### 3.8 Stomatal responses and ultrastructure of leaves

**Figure 4:**
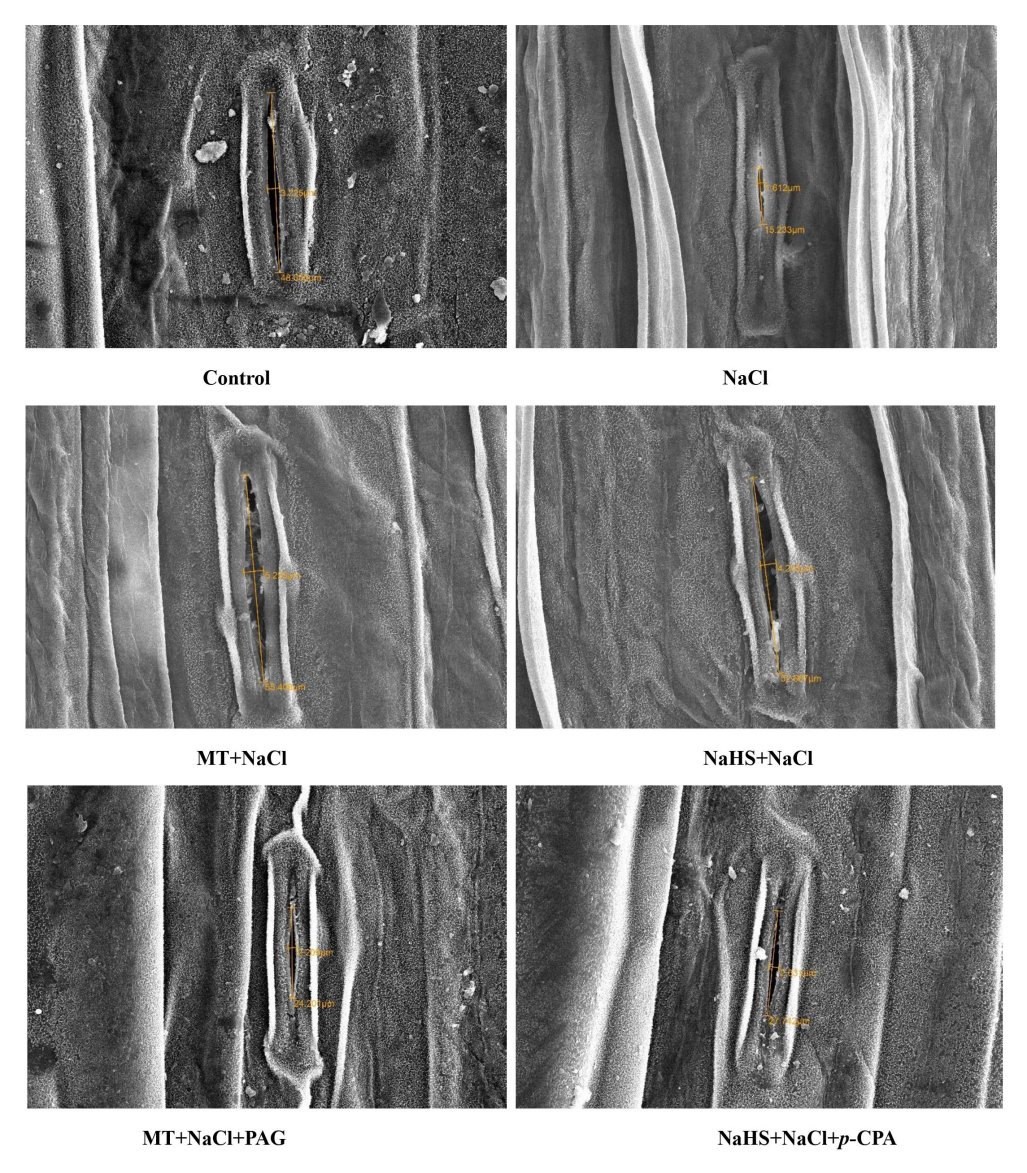
Stomatal response of wheat (*Triticum aestivum L.* cv. GOAL) at 30 days after sowing using scanning electron microscopy (SEM) at 1000x, under control; 100 mM NaCl, 100µM MT +NaCl; 200 µM NaHS +NaCl; MT +NaCl+ 1mM PAG, NaHS+NaCl+10μM *p*-CPA. MT: melatonin; NaHS: sodium hydrosulfide (H_2_S donor); PAG: DL-propargylglycine (H_2_S synthesis inhibitor); *p*-CPA: *p*-chlorophenyl alanine (melatonin synthesis inhibitor).

Conducting SEM analysis, we observed stomatal responses under varied treatments (**Figure 4**). Stomata exhibited maximum opening under control conditions, but salt stress caused a reduction in diameter. Application of MT and NaHS individually improved stomatal aperture, with MT showing a more pronounced effect. However, plants treated with MT + NaCl + PAG displayed damaged stomatal apertures and maximum distortion compared to NaHS + NaCl + *p*-CPA. This suggests that MT mediates its effects through H_2_S, regulating stomatal behavior under salt stress.

**Figure 5:**
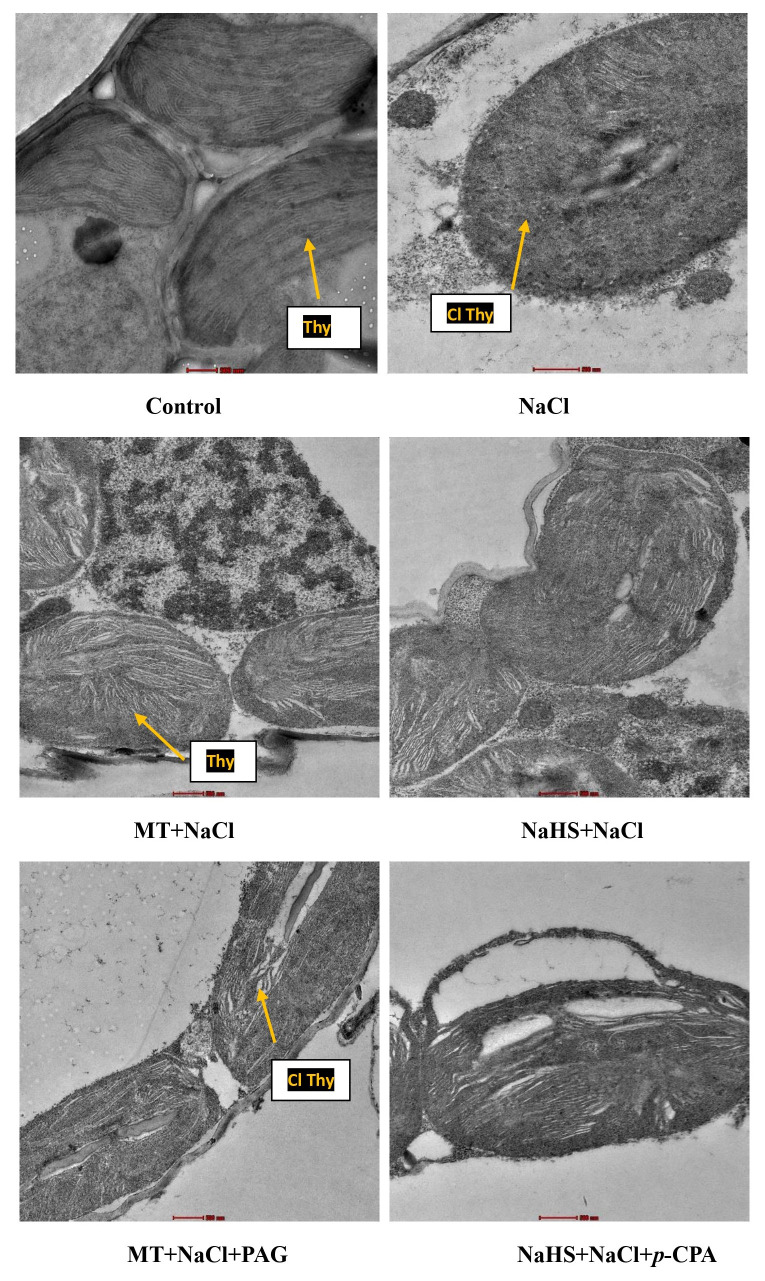
Chloroplastic structures of wheat (*Triticum aestivum L.* cv. GOAL) at 30 days after sowing using transmission electron microscopy (TEM) at scale 500 nm, under control; 100 mM NaCl, 100µM MT +NaCl; 200 µM NaHS +NaCl; MT +NaCl+ 1mM PAG, NaHS+NaCl+10μM *p*-CPA. MT: melatonin; NaHS: sodium hydrosulfide (H_2_S donor); PAG: DL-propargylglycine (H_2_S synthesis inhibitor); *p*-CPA: *p*-chlorophenyl alanine melatonin synthesis inhibitor); Thy, thylakoids; Cl Thy, damaged thylakoid.

Similarly, examining chloroplastic structure through TEM revealed damage due to salinity (**Figure 5**). MT showed a positive effect by maintaining the integrity of the chloroplastic structure, followed by NaHS under salt stress. Application of inhibitors indicated that plants treated with MT + NaCl + PAG exhibited more chloroplastic damage compared to those treated with NaHS + NaCl + *p*-CPA. This suggests that the inhibition of endogenous H_2_S synthesis by PAG affects MT responses under salt stress. This implies that both MT and H_2_S are required to maintain chloroplastic structure under salt stress, with MT showing a more beneficial effect and H_2_S is required by MT for its action on maintaining chloroplastic structures.

### 3.9 Total soluble sugars, total non-structural carbohydrates, and starch content

**Figure 6:**
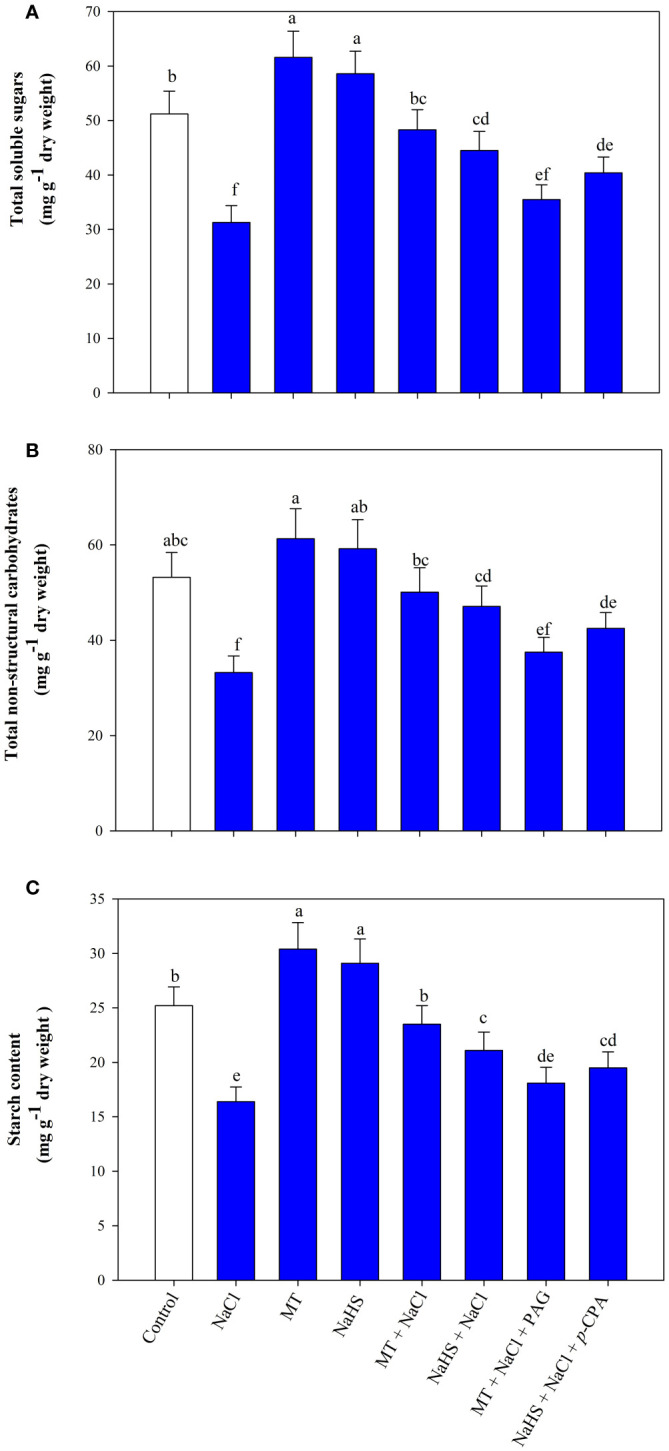
Content of total sugars **(A)**, total non-structural carbohydrates **(B)**, and starch **(C)** of wheat (*Triticum aestivum* L. cv. GOAL) at 30 days after sowing (DAS). Plants were treated with 100 µM melatonin (MT) or 200 µM sodium hydrosulfide (NaHS; H_2_S donor) in the presence or absence of 100 mM NaCl or were treated with 1mM DL-propargylglycine (PAG; H_2_S synthesis inhibitor) with MT and NaCl or with 10 μM *p*-chlorophenylalanine (*p*-CPA, melatonin synthesis inhibitor) with NaHS and NaCl. The foliar treatments of MT, NaHS, PAG, and *p*-CPA were given at 15 DAS. The data is displayed as the mean of treatments ± standard error (n = 4). Values with identical letters showed no significant differences according to the LSD test at a significance level of *p*< 0.05.

Salinity decreases the soluble sugars, TSC, and starch content by 38.8%, 37.5%, and 34.9% compared to the control group (**Figures 6A-C**). MT and NaHS showed a positive effect by enhancing their content under both stressed and unstressed conditions. Under stress, MT application increased soluble sugars, TSC, and content by 54.3, 50.9 and 43.2% and NaHS by 42.1, 41.8 and 28.6%, respectively, compared to only salt-stressed plants. Exogenously applied MT notably alleviated the detrimental effects of salinity stress on carbohydrate metabolism characteristics more effectively than NaHS. Yet, the effects of MT and NaHS on these aspects were significantly reduced by inhibitors, specifically PAG and *p*-CPA. Remarkably, a more substantial adverse impact was evident in the treatment involving MT + NaCl + PAG in comparison to NaHS + NaCl + *p*-CPA. This implies that MT affects carbohydrate metabolism through H_2_S.

### 3.10 Calvin cycle enzymes

**Figure 7:**
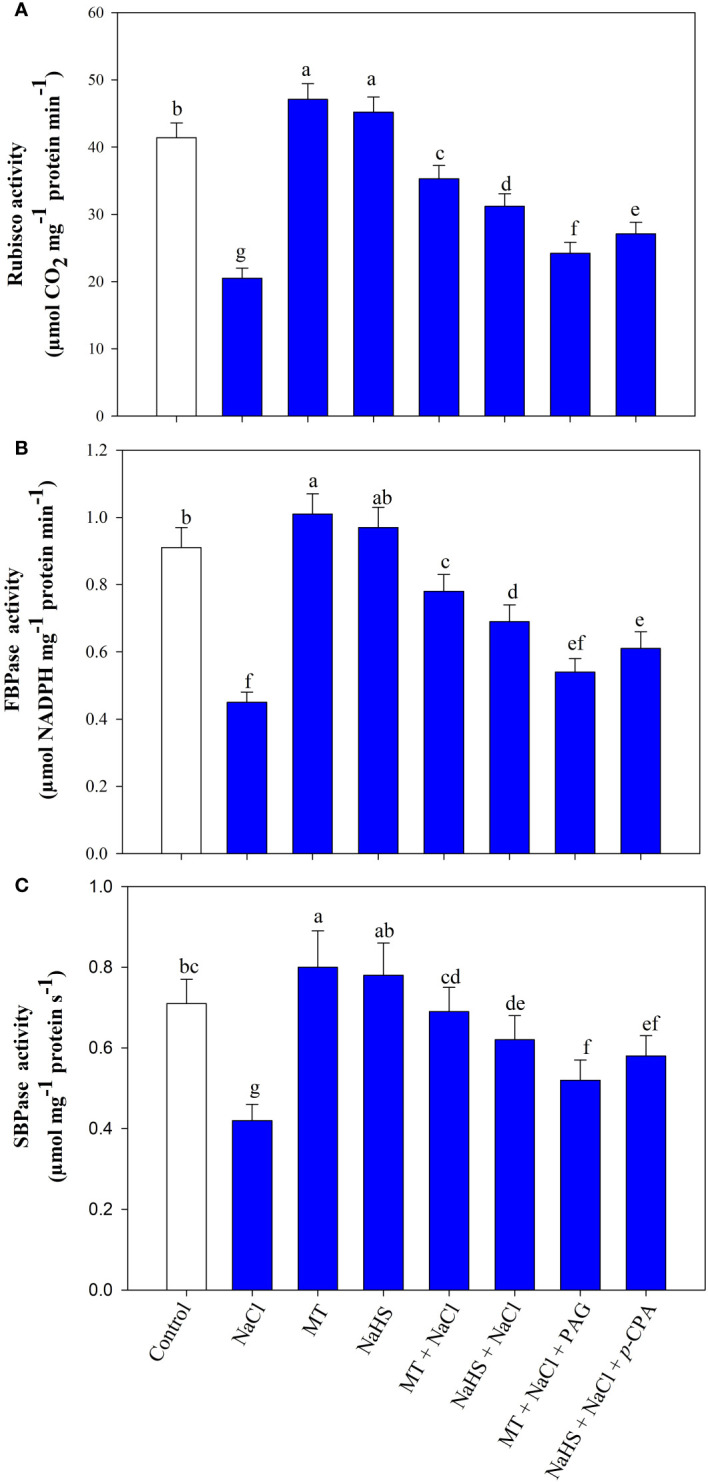
Activity of Rubisco **(A)**, fructose-1,6-bisphosphatase (FBPase) **(B)**, sedoheptulose-1,7-bisphosphatase (SBPase) **(C)** of wheat (*Triticum aestivum* L. cv. GOAL) at 30 days after sowing (DAS). Plants were treated with 100 µM melatonin (MT) or 200 µM sodium hydrosulfide (NaHS; H_2_S donor) in the presence or absence of 100 mM NaCl or were treated with 1mM DL-propargylglycine (PAG; H_2_S synthesis inhibitor) with MT and NaCl or with 10 μM *p*-chlorophenylalanine (*p*-CPA, melatonin synthesis inhibitor) with NaHS and NaCl. The foliar treatments of MT, NaHS, PAG, and *p*-CPA were given at 15 DAS. The data is displayed as the mean of treatments ± standard error (n = 4). Values with identical letters showed no significant differences according to the LSD test at a significance level of *p*< 0.05.

Plants under salinity showed a considerable reduction in the activity of Rubisco by 50.5%, fructose-1,6-bisphosphatase (FBPase) by 50.1%%, sedoheptulose-1,7-bisphosphatase (SBPase) by 40.8% compared to control (**Figures 7A-C**). Individual application of MT and NaHS under unstressed and stressed conditions improved the activity of these enzymes. Under salt stress, the activity of rubisco, FBPase, and SBPase was enhanced with the treatment of MT by (72.1%, 69.5%, and 64.2%) and NaHS by (52.2%, 50.0%, and 47.6%) in comparison to only salt-stressed plants. Conversely, the application of inhibitors, PAG and *p*-CPA, reduced enzymatic activity, and maximum reduction of rubisco, FBPase, and SBPase activity was observed in treatment MT +NaCl +PAG. Thus, results showed that MT has a more beneficial effect compared to H_2_S, but somehow, H_2_S is involved in melatonin-mediated protection of Calvin cycle enzymes.

### 3.11 Yield

Salinity stress adversely affected the plant’s yield characteristics (**Table 3**), resulting in a 19.0% reduction in spike length, a 27.4% decrease in spikelets per spike, a 32.6% drop in grains per spike, and a 38.7% decline in the weight of 1000 grains compared to control plants. Conversely, treatment with MT and NaHS under non-stress conditions enhanced these parameters compared to the control. Furthermore, when subjected to NaCl stress, the application of either MT or NaHS effectively mitigated the negative effects of stress. This was evidenced by improvements in spike length (20.4% and 16.3%), spikelets per spike (31.4% and 24.1%), grains per spike (41.5% and 31.4%), and 1000-grain weight (39.7% and 37.1%) compared to plants exposed solely to salt stress. The findings elucidate the positive role of MT and NaHS in enhancing agronomic traits under stressful conditions. However, it is noteworthy that MT demonstrated greater efficacy than NaHS. Intriguingly, the introduction of PAG in conjunction with MT + NaCl resulted in a more pronounced reduction in yield-related characteristics compared to plants treated with NaHS + NaCl + *p*-CPA. This suggests that PAG can counteract the positive effects induced by MT under stress conditions.

**Table 3:** Spike length, spikelet per spike, grain per spike, and 1000 grain weight of wheat (*Triticum aestivum* L. cv. GOAL) at maturity of crop.

Treatments	Spike length (cm)	Spikelet per spike	Grain per spike	Grain weight (g)
Control	12.1 ± 0.89b	17.1 ± 1.03b	53.6 ± 4.0b	44.6 ± 2.07bc
**NaCl**	9.8 ± 0.35d	12.4 ± 0.84e	36.1 ± 2.4e	27.3 ± 1.32g
**MT**	14.4 ± 0.95a	20.9 ± 1.34a	66.3 ± 4.5a	51.9 ± 2.36a
**NaHS**	14.1 ± 0.92a	20.3 ± 1.28a	63.4 ± 4.1a	48.8 ± 2.32ab
**MT + NaCl**	11.8 ± 0.88bc	16.3 ± 1.01b	51.1 ± 3.8b	39.7 ± 1.74cd
**NaHS + NaCl**	11.4 ± 0.87bc	15.4 ± 0.98bc	47.5 ± 3.2bc	37.1 ± 1.74de
**MT +NaCl + PAG**	10.4 ± 0.29cd	13.1 ± 0.61de	39.5 ± 2.0de	31.5 ± 1.21fg
**NaHS +NaCl + *p*-CPA**	10.7 ± 0.54bcd	14.4 ± 0.72cd	44.3 ± 2.2cd	33.6 ± 1.28ef

Plants were treated with 100 µM melatonin (MT) or 200 µM sodium hydrosulfide (NaHS; H_2_S donor) in the presence or absence of 100 mM NaCl or were treated with 1mM DL-propargylglycine (PAG; H_2_S synthesis inhibitor) with MT and NaCl or with 10 μM *p*-chlorophenylalanine (*p*-CPA, melatonin synthesis inhibitor) with NaHS and NaCl. The foliar treatments of MT, NaHS, PAG, and *p*-CPA were given at 15 DAS. The data is displayed as the mean of treatments ± standard error (n = 4). Values with identical letters showed no significant differences according to the LSD test at a significance level of *p*< 0.05.

### 3.12 Principal component analysis

**Figure 8:**
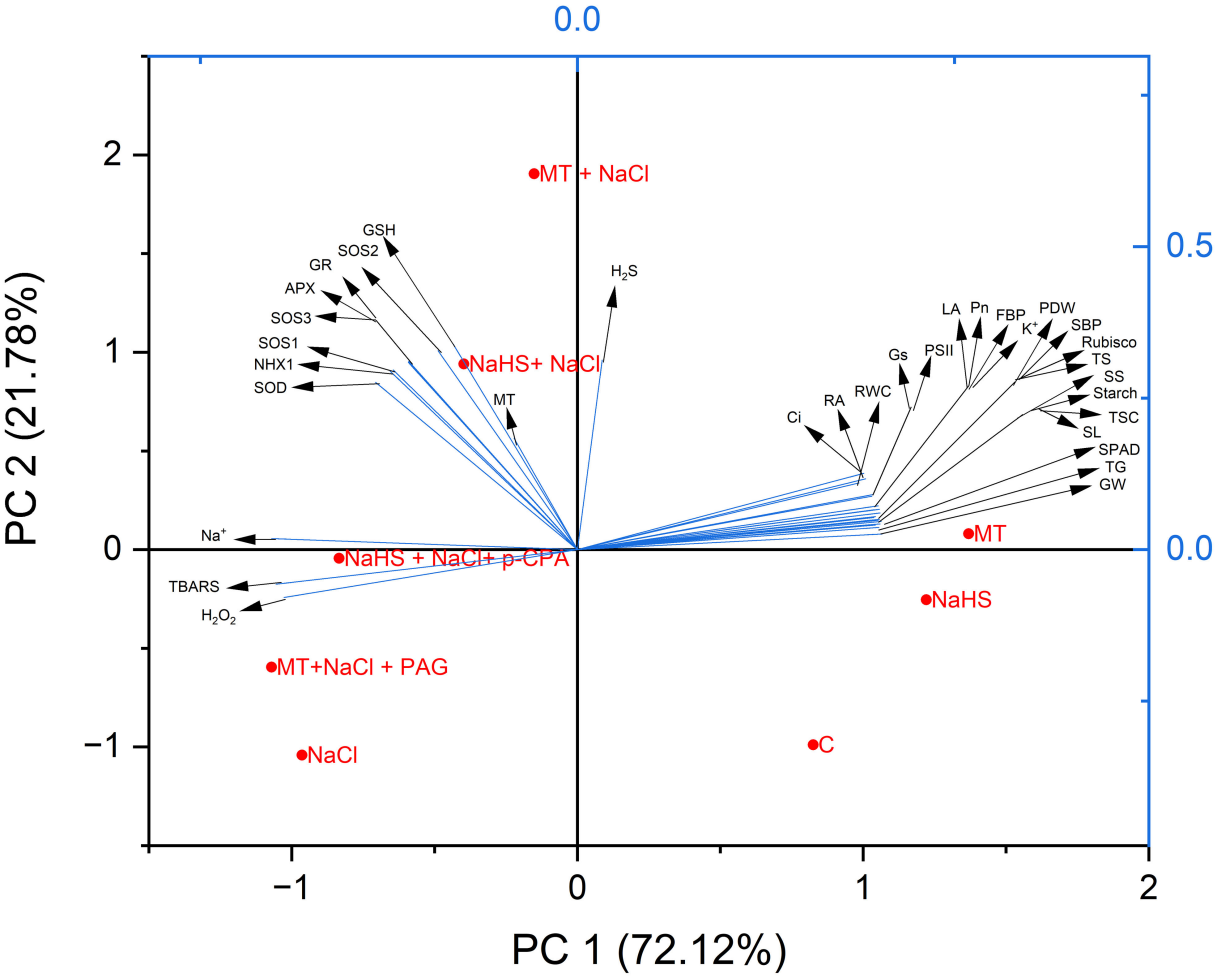
Principal Component Analysis (PCA) biplots depict the connections between various treatments and variables in wheat (*Triticum aestivum* L.) cv. GOAL subjected to diverse growth conditions such as: control; NaCl (100mM); MT((100µM) +NaCl; NaHS (200 µM) +NaCl; MT +NaCl +PAG(1mM); NaHS+NaCl+p-CPA(10μM). MT: Melatonin; NaHS: sodium hydrosulfide (H_2_S donor); PAG: DL-propargylglycine(H_2_S synthesis inhibitor); *p*-CPA: p-chlorophenyl alanine melatonin synthesis inhibitor). The variables included H_2_O_2_ (hydrogen peroxide), TBARS (thiobarbituric acid reactive substances), Na^+^(Sodium content), K^+^(potassium content), SOD (superoxide dismutase), APX (ascorbate peroxidase), GR (glutathione reductase) activity, the content of GSH(glutathione), gene expression of (*SOS1, SOS2, SOS3* and *NHX1*), contents of MT(melatonin, H_2_S (hydrogen sulfide), Pn (net photosynthesis), Gs (stomatal conductance), Ci (intercellular CO_2_ concentration), SPAD value, PSII (maximum efficiency of PSII), RA (root activity), RWC (relative water content), LA(leaf area), PDW(plant dry weight), the activity of Calvin cycle enzyme rubisco; FBP(fructose-1,6-bisphosphatase), SBP (sedoheptulose-1,7-bisphosphatase), the content of TS(total soluble sugars, TSC(total non-structural carbohydrates), starch, and agronomic characters SL(spike length), SS(spikelet per spike), TG(grain per spike), GW(weight of 1000 grain).

Principal Component Analysis (PCA) assessed data variability and elucidated the interrelationships among different treatments and parameters. The two primary components, PC1 and PC2, collectively accounted for 93.9% of the observed data variability in response to various treatments (**Figure 8**). Specifically, PC1 contributed significantly, representing 72.12% of the total variation, while PC2 accounted for 21.78%. The resulting biplot delineated three distinct clusters. Notably, photosynthetic, growth, and agronomic parameters (gas-exchange parameters, activity of Calvin cycle enzymes, root activity, RWC, total sugars, starch, TSC content, leaf area, PDW, spike length, spikelet per spike, total grain per spike, and 1000 grain weight), exhibited a cohesive clustering, indicative of a positive relationship. This cluster was closely associated with control, MT, and NaHS treatment. Conversely, oxidative stress indicators, including H_2_O_2_, TBARS, and Na^+^ content, formed a separate cluster. Notably, this cluster showed proximity to NaCl stress treatment, implying a negative correlation with photosynthetic, growth, and agronomic parameters. Antioxidants (SOD, APX, GR, and GSH), Na^+^-ion transporter gene (*SOS1, SOS2, SOS3*, and *NHX1*), and the content of MT and H_2_S were positioned amidst oxidative stress parameters and photosynthetic and growth parameters. Remarkably, they exhibited proximity to MT +NaCl and NaHS +NaCl treatments, suggesting their pivotal role in mitigating salt stress. Additionally, treatments MT +NaCl +PAG and NaHS +NaCl + *p*-CPA were found to be closely associated with NaCl, indicating that both hormones are synergistically required for enhanced efficacy in mitigating salt stress.

## 4 Discussion

Excessive salinity induces osmotic stress in plants, leading to membrane damage, ROS accumulation, and ion imbalance, reducing growth and crop yield. Plants have evolved diverse mechanisms to cope with different environmental challenges. MT recognized as a novel plant growth regulator, is believed to play a role in various responses to biotic and abiotic stresses. In this study, we explored the relative effectiveness of MT and H_2_S and the interplay of these signaling molecules in mitigating salt-induced effects in wheat. The response of plants to 100 µM MT treatments was most effective, followed by 200 µM NaHS. This highlights their potential to enhance salt stress tolerance in wheat plants. Notably, our findings suggest that MT’s positive impact on wheat under salt stress is facilitated through H_2_S, revealing a potential synergy between these compounds in promoting plant resilience.

In response to salt stress, plants experience elevated levels of ROS, leading to oxidative damage, as indicated by the increased TBARS content. The application of MT and NaHS effectively reduced H_2_O_2_ and TBARS levels, presenting a promising strategy to ameliorate oxidative damage caused by salt stress. Notably, MT treatment demonstrated superior efficacy in alleviating salt-induced oxidative damage compared to NaHS, evidenced by decreased H_2_O_2_ and TBARS content. The delicate balance between ROS scavenging and generation in plants involves the activation of antioxidant enzymes such as SOD, POX, CAT, APX, and GR under stress conditions (Zhan et al., 2019). Similarly, under salt stress, the enhanced activity of SOD, APX, and GR was observed in wheat leaves, and the application of MT and NaHS further augmented the activity of these enzymes, along with an increase in GSH content. These results emphasized that MT treatment enhanced the antioxidant defense machinery more effectively than NaHS. As previously observed, MT’s broad-spectrum antioxidant defense capabilities, are demonstrated through efficient ROS scavenging, making it a potential antioxidant compared to the other non-enzymatic antioxidants (Moustafa-Farag et al., 2020). Additionally, the study explored the interplay between melatonin and H_2_S in alleviating salt-induced oxidative stress using MT +NaCl +PAG and NaHS +NaCl +*p*-CPA combinations. We found that H_2_O_2_ and TBARS content was high in leaves treated with MT +NaCl +PAG, revealing that the maximum alleviation of salt stress by MT occurred through stimulation of the antioxidant defense machinery via H_2_S. Previous research has shown that MT upregulates antioxidant enzyme expression and activity, mitigating the adverse effects of salt stress in various crops such as tomato, and sorghum (Yildrim et al., 2023; Nie et al., 2023). Similarly, studies have demonstrated that H_2_S improves the activity of antioxidant enzymes, such as SOD, POD, CAT, APX, and GR in rice and cucumber plants under salt stress (Mostofa et al., 2015; Turan et al., 2022). Moreover, the modulation of endogenous H_2_S homeostasis and L-Cysteine Desulfhydrase activity by exogenous MT, along with the effective regulation of antioxidant enzyme activity and enhancement of the AsA–GSH cycle metabolism by H_2_S (Lai et al., 2014; Mukherjee and Bhatla, 2021) further support the intricate crosstalk between these two signaling molecules in response to abiotic stress across different crops (Iqbal et al., 2021; Mukherjee and Bhatla, 2021; Haghi et al., 2022).

In salt stress, the influx of excessive Na^+^ and Cl^–^ ions into plants increases their accumulation at elevated concentrations, leading to ion toxicity. Notably, owing to their similar ionic radius and hydration energy, K^+^ and Na^+^ exhibit a pronounced antagonistic relationship. Consequently, excessive Na^+^ often induces K^+^ leakage, impacting various physiological and metabolic processes in plants (Yan et al., 2020; Nie et al., 2023). The increased soil osmotic potential resulting from salinity hinders the plant’s ability to absorb adequate water from the soil, consequently enhancing osmotic stress (Shahnaz et al., 2021). Both salt-induced osmotic stress and ion toxicity can provoke an overproduction of ROS in plant cells, leading to membrane instability, electrolyte leakage, and increased lipid peroxidation in cell membranes, affecting plant growth (Yan et al., 2020; Jiang et al., 2021). The Salt Overly Sensitive (SOS) pathway maintains ion homeostasis and facilitates plant adaptation to salinity stress (Younis and Mansour, 2023). Within this pathway, SOS2, a protein kinase, serves as a central hub and rapidly activates its kinase activity in response to salinity stress. This activation leads to the formation of a complex with SOS3 on the cell membrane, subsequently triggering the activation of the SOS1 antiporter. SOS1 is pivotal in excluding Na^+^ to outer spaces and regulating long-distance Na^+^ transport from roots to shoots (Chen et al., 2023). Moreover, tonoplast Sodium/hydrogen antiporters (NHXs) are crucial in regulating cellular cation homeostasis. The absorption of K^+^ into vacuoles and the sequestration of Na^+^ affect the stomatal function and preserve cellular pH (Bassil and Blumwald, 2014). In our study, wheat plants exhibited reduced RWC and K^+^/Na^+^ ratio under salt stress conditions due to a decrease in shoot K^+^ content and an increase in Na^+^ content. Notably, MT and NaHS exhibit the capacity to regulate Na^+/^K^+^ ion balance in crop plants by increasing the expression of K^+^ channel genes (*SKOR, AKT1*), SOS-pathway gene (*SOS1-3*), and plasma membrane H-ATPases, thereby enhancing salt tolerance in tomato, bittermelon, and cucumber (Sheikhalipour et al., 2022; Ghorbani et al., 2023; Luo et al., 2023). Consistent with these findings, our study reveals that the application of MT and H_2_S enhanced the expression of the *SOS1. SOS2, SOS3*, *NHX1* gene, and RWC maintained Na^+/^K^+^ homeostasis by reducing Na^+^ and increasing K^+^ content. Notably, MT exhibits a more significant effect compared to H_2_S. Upon application of inhibitors in the treatment, such as MT +NaCl +PAG and NaHS +NaCl +*p*-CPA, MT’s efficacy is diminished in the presence of PAG. Conversely, a reduction in the expression of SOS-pathway genes, *NHX1*, sodium, potassium content, and RWC was observed with NaHS in the presence of p-CPA, though to a lesser extent. This suggests that MT maintains the Na^+^/K^+^ ratio by influencing the expression of SOS-pathway and *NHX1* genes through H_2_S. Similar findings have been reported in tomatoes (Mukherjee and Bhatla, 2021), where H_2_S is implicated in the MT-mediated regulation of the Na^+^/K^+^ antiport system. The improved RWC may be attributed to enhanced K^+^ levels, which modulate the expression of water channel protein genes and contribute to maintaining water balance (Gao et al., 2022). Additionally, the recorded increase in RWC could result from the participation of MT and H_2_S in influencing stomatal behavior. This effectively manages the opening and closure of stomata, preventing excessive water loss from leaves (Lisjak et al., 2010; Xu et al., 2010). Thereby maintaining RWC under stress.

Roots play a vital role as an essential organ in the absorption and conversion of biologically active substances throughout the growth and maturation of crops. Additionally, they are the primary organ in perceiving and responding physiologically to adverse stress conditions (Nie et al., 2023). Root activity, encompassing root growth, vigor, and physiological functioning, directly influences crop growth, nutritional status, and yield (Wu et al., 2017). Existing literature suggests that water status, plant growth stage, and soil conditions can impact root activity (Wu et al., 2017). Our study revealed a reduction in root activity under salt stress, likely attributed to osmotic stress and disruptions in nutrient balance. This decline was mitigated upon applying exogenous MT and NaHS, aligning with the findings in *Carex leucochlora* (Ren et al., 2022) and *Malus hupensis* (Li et al., 2020) under salt stress. Interestingly, the co-application of the H_2_S biosynthesis inhibitor PAG with MT +NaCl led to a more pronounced reduction in root activity compared to the use of MT biosynthesis inhibitor *p*-CPA in combination with NaHS +NaCl. This discrepancy may be attributed to MT mediating its signal to alleviate osmotic stress through H_2_S, which was impeded in the presence of PAG as the production of H_2_S was diminished.

Under NaCl stress, there is an observed increase in endogenous MT and H_2_S content, as documented in previous studies (ElSayed et al., 2020; Mukherjee and Bhatla, 2021). The external supplementation of MT further amplifies the evolution of H_2_S triggered by salt stress, attributed to the increased activity of L/D-cysteine desulfhydrase (Mukherjee and Bhatla, 2021). Interestingly, the addition of NaHS under stress did not significantly alter MT content compared to plants exposed only to salt stress. Conversely, when PAG was applied with MT +NaCl, a notable decrease in H_2_S content occurred, resulting in a more substantial reduction in photosynthesis, growth, and yield parameters. In the treatment MT +NaCl +PAG, although MT content remained high, the drastic reduction in H_2_S content led that MT failed to mediate its positive response in alleviating salinity stress. This finding aligns with the report published earlier (Siddiqui et al., 2021; Sun et al., 2021), where the supplementation of hypotaurine to salt-stressed tomato and cucumber plants, respectively, with MT, reduced H_2_S levels, leading to disruption in ion homeostasis and photosynthesis parameters. Similarly, the application of *p*-CPA under salt stress reduced MT, which ultimately reduced H_2_S content, as MT was involved in enhancing the activity of L/D-cysteine desulfhydrase under stress, as observed in a prior report on cucumber grown under salt stress (Sun et al., 2021).

Photosynthesis is crucial for sustaining plant growth and development, especially in optimal and challenging conditions (Muhammad et al., 2021). The decline in photosynthetic activity under salt-induced stress can be attributed to factors affecting stomatal and non-stomatal functions. Stomatal limitations involve a reduction in stomatal conductance, hindering the influx of CO_2_ from the air into mesophyll cells and consequently impacting the overall photosynthetic rate (Yan et al., 2023). In our investigation, the imposition of salinity resulted in a significant reduction in net photosynthesis, stomatal conductance, and intercellular CO_2_ concentration. Notably, the application of either MT or NaHS demonstrated a notable improvement in these critical gas-exchange parameters, underscoring their positive influence on stomatal behavior. This enhancement was further evident in the scanning electron microscopy (SEM) images of leaves, which displayed an improved stomatal aperture length and breadth in comparison to leaves subjected solely to salinity stress. Interestingly, the impact was more pronounced with MT compared to NaHS. Aligning with a growing body of evidence supporting the beneficial effects of MT and NaHS application across various plant species such as tomato, sorghum, cucumber, and wheat, our findings are consistent with previous studies (Yildrim et al., 2023; Ke et al., 2018; Luo et al., 2023; Nie et al., 2023). Melatonin, in particular, demonstrated an enhancement in stomatal characteristics by increasing length, width, and opening, while H_2_S positively influenced stomatal density and aperture (Duan et al., 2015; Jiang et al., 2021). In the current study, the application of PAG with MT +NaCl resulted in a diminished positive effect on gas-exchange parameters and stomatal behavior compared to the treatment involving NaHS +NaCl +*p*-CPA. This observation suggests that H_2_S has MT-mediated effects on gas exchange and stomatal factors, highlighting the intricate interplay between these signaling molecules in response to salinity stress.

Non-stomatal limiting factors are crucial in diminishing photosynthesis by impeding carbon assimilation. This inhibition involves reduced light-harvesting complex energy uptake, diminished photosystem activity, and decreased enzyme activity essential for carbon fixation, leading to the underutilization of CO_2_ (Yan et al., 2023). Under salinity stress, a decline in the SPAD value in wheat leaves was observed, likely attributed to the disruption of chloroplast structure. This disruption hampers enzymes involved in chlorophyll synthesis and enhances the activity of the chlorophyll-degrading enzyme chlorophyllase due to the accumulation of Na^+^ and Cl^−^ (Raju and Prasad, 2023). In our study, the exogenous application of either MT or NaHS under salt stress significantly increased chlorophyll content, indicating a protective role in preserving thylakoid membrane integrity and overall chloroplast structure. The ultrastructure of chloroplasts revealed a more pronounced effect of MT compared to NaHS in enhancing chlorophyll synthesis and maintaining chloroplast structure under stress. Transcriptome studies suggest that MT downregulates the expression of genes responsible for chlorophyll-degrading enzymes. Similar studies on maize plants supplemented with MT under drought stress showed well-preserved chloroplast structures, and H_2_S was found to enhance chloroplast biogenesis by increasing the quantity of grana lamellae (Rizwan et al., 2019; Ahmad et al., 2022). Moreover, these treatments enhance antioxidants, protecting chloroplast membranes and stabilizing enzymes involved in photosynthetic pigment synthesis and the carbon assimilation cycle (Raju and Prasad, 2023).

Photosynthetic electron transport is essential for maintaining proper plant growth, development, and stress responses. Under salt stress, inadequate dissipation mechanisms expose plants to excessive energy, impacting the photosystem (Yan et al., 2021). The Fv/FM ratio is a reliable indicator of photosynthetic inhibition, and our results demonstrate that both MT and NaHS alleviate the lower Fv/FM under salt stress. This suggests that both compounds mitigate the adverse effects of salt stress on Photosystem II efficiency, potentially by stabilizing PSII proteins, promoting electron transfer, and improving chlorophyll and carotenoid content under stress (Tang et al., 2020; Yan et al., 2021).

Rubisco, a key enzyme in the Calvin cycle, plays a pivotal role in regulating photosynthesis as it is involved in the first step of carboxylation (Hameed et al., 2021). Other enzymes, sedoheptulose-1,7-bisphosphatase (SBPase) and fructose-1,6-bisphosphatase (FBPase) are integral to the cycle’s progression. FBPase plays a pivotal role in the regeneration phase of the cycle and the synthesis of starch, functioning as an intermediate at a critical branching point (Kossmann et al., 1994). Similarly, SBPase influences whether carbon is recycled for ribulose-1,5-bisphosphate (RuBP) regeneration or exits the cycle for starch biosynthesis, making it vital for maintaining the flow of carbon sources (Chao et al., 2023). Under salt stress in our study, there was a substantial reduction in the activity of these enzymes, impacting carbon fixation and carbohydrate synthesis. However, the application of MT and NaHS exerted a protective effect on the activities of these enzymes, enhancing photosynthesis under salt stress. Consistent with our findings, studies on green beans, cabbage, and kiwi revealed that exogenous MT and H_2_S regulate the expression of *RbcL* and *RbcS* genes, suggesting a regulatory role for these compounds in Rubisco structure and function under salt and drought stress conditions (Liang et al., 2019; ElSayed et al., 2021; Zhang et al., 2023). Also, MT application in kiwi under drought stress modulates the carbon fixation by upregulating the expression of Rubisco, fructose-1,6-bisphosphatase, sedoheptulose-1,7- bisphosphatase, fructose-bisphosphate aldolase, and ribose 5-phosphate isomerase (Liang et al., 2019). Moreover, proteomics studies by Wei et al. (2021) demonstrated the upregulation of proteins such as phosphoglycerate kinase and sedoheptulose-1,7-bisphosphatase upon NaHS treatment. This upregulation facilitated the recovery of regular Calvin cycle activity in rice seedlings under salt stress. In summary, the protective effects of MT and NaHS on the activity and expression of the Calvin cycle enzymes underscore their crucial role in sustaining carbon flow, promoting efficient photosynthesis, and mitigating the adverse impacts of salt stress on plant metabolic processes. Further in this study, the application of PAG with MT +NaCl diminished the positive effects of MT on chlorophyll content, chloroplast ultrastructure, PSII efficiency, and Calvin cycle enzyme activity more than the treatment involving NaHS +NaCl+ *p*-CPA. This suggests that in the presence of PAG, MT cannot mediate its response against salt stress. Conversely, H_2_S, even in the presence of *p*-CPA, mediated its response, although not as effectively as without *p*-CPA under stress. This implies that MT has a more profound effect on improving photosynthesis under stress compared to H_2_S, but MT requires H_2_S to mediate its response. Consistent with this, previous reports have highlighted the crosstalk between MT and H_2_S in enhancing photosynthesis in wheat under heat stress and reducing oxidative stress through increased antioxidant enzymes and carbohydrate metabolism (Iqbal et al., 2021). Additionally, H_2_S is involved in MT-mediated improvements in chlorophyll content and glutathione levels, reducing oxidative burst and enhancing plant photosynthesis under arsenic stress (Kaya et al., 2022).

Various pathways of carbohydrate metabolism in plants are likely to undergo modulation in response to diverse abiotic stresses. A crucial aspect of abiotic stress tolerance in plants involves the conversion between starch and sugars in source organs (Thalmann and Santelia, 2017). The impact of NaCl on plants manifests in a reduction of Calvin cycle enzymes, accompanied by decreases in starch, soluble sugars, and total non-structural carbohydrate (TSC) content. This phenomenon may be attributed to the diminished activity of starch biosynthesis enzymes and invertase (Rosa et al., 2009; Iqbal et al., 2021; Khan et al., 2021). However, the application of either MT or NaHS demonstrates positive effects by enhancing the content of soluble sugars and starch in wheat leaves under salt stress. Nevertheless, the impact of MT was more pronounced, resulting in significantly higher levels of soluble sugars, starch, and TSC compared to NaHS. The enhanced levels of soluble sugars contribute to improved defense against stress responses, enhance RWC, and lead to osmotic tolerance. Also, reports showed that they play a crucial role in various metabolic processes, acting as signaling molecules to regulate the expression of numerous photosynthesis-related genes (Fatma et al., 2023). Interestingly, the combined application of PAG with MT +NaCl reverses the positive effect of MT more effectively than the application of *p*-CPA in combination with NaHS +NaCl. This implies that both hormones are necessary to alleviate the adverse effects of salinity stress on carbohydrate metabolism, and the positive impact of MT is mediated through H_2_S. In line with our findings, Iqbal et al. (2021) reported that MT, via H_2_S, enhances the activity of ADP-glucose phosphorylase and invertase in wheat under heat stress, resulting in improved starch and soluble sugar levels. Khan et al. (2021) found that exogenous K^+^ in an H_2_S-dependent manner partially boosts starch accumulation in the roots of tomato seedlings stressed by NaCl, influencing starch biosynthesis and accumulation. This rise in starch is accompanied by a partial reduction in sucrose levels in the stressed tomato seedling roots. Additionally, increased invertase activity and higher total soluble sugars accumulation suggest improved sucrose metabolism and the formation of hexose sugars during K^+^ supplementation under NaCl stress.

**Figure 9:**
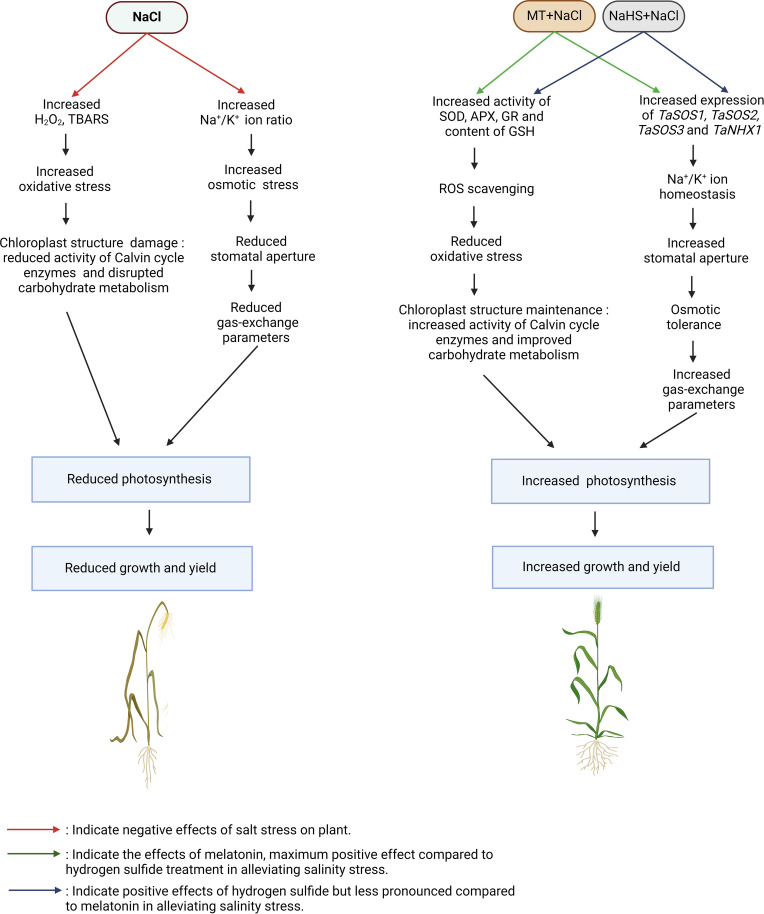
A model illustrating the role of melatonin and H_2_S in the alleviation of salt stress in wheat (*Triticum aestivum* L.). NaCl led to a reduction in photosynthesis, growth, and yield of plants compared to control. Both MT and H2S help in mitigating the negative effects of salinity, but MT has a more pronounced effect compared to H2S. MT: Melatonin; NaHS: sodium hydrosulfide (H_2_S donor); H_2_O_2_: hydrogen peroxide; TBARS: thiobarbituric acid reactive substances; Na^+^: sodium ion; K^+^: potassium ion; SOD: superoxide dismutase; APX: ascorbate peroxidase; GR: glutathione reductase; GSH: reduced glutathione; SOS1/2/3: salt overly sensitive; NHX1: Na^+^/H^+^ exchanger.

The deleterious impact of salinity on plant photosynthesis reduces leaf area, plant dry weight, and key yield parameters such as spike length, spikelets per spike, grains per spike, and the weight of 1000 grains in wheat. Salinity disrupts ionic homeostasis and osmotic balance, leading to the generation of ROS, consequently diminishing photosynthesis, carbohydrate metabolism, and nitrogen metabolism. This cascade of events ultimately hampers plant growth and yield (Abbas et al., 2013; Elbagory, 2023; Khan et al., 2023). Remarkably, the application of both MT and NaHS exogenously significantly enhances all evaluated growth and yield parameters. This aligns with previous findings indicating that melatonin application enhances crops such as tomato, wheat, and soybean growth under salinity conditions (Wei et al., 2015; Zafar et al., 2019; Khan et al., 2023). Similarly, H_2_S has been reported to increase the growth and yield of various crops, including wheat, strawberry, and broccoli (Bahmanbiglo and Eshghi, 2021; Kumari et al., 2023; Shalaby et al., 2023). The positive effects observed with MT and NaHS are attributed to their ability to scavenge ROS by enhancing the antioxidant machinery, maintaining Na^+^/K^+^ homeostasis by enhancing expression of genes of SOS-pathway and *NHX1* and maintaining osmotic tolerance by enhancing RWC. This, in turn, protects plant photosynthesis, ultimately contributing to enhanced growth and yield. Notably, in this study, the application of PAG with MT +NaCl has a more significant negative impact on growth and yield compared to the addition of p-CPA with NaHS +NaCl. This difference is attributed to the H_2_S-mediated responses of MT in mitigating salinity stress. A model illustrating the importance of MT and H_2_S in enhancing tolerance to salinity stress is depicted in **Figure 9**.

## 5 Conclusion

Plant photosynthesis, growth, and yield are all negatively impacted by salt stress, which disrupts ion homeostasis and causes oxidative stress, as evidenced by the PCA plot. Salt-induced ramifications in wheat plants have been effectively mitigated by MT and H_2_S. The enhanced photosynthetic and growth attributes that result from MT’s direct antioxidant action increased antioxidant enzyme activity, thus maintaining chloroplast structure. Further, the reduction of Na^+^ content through upregulation of *SOS1, SOS2, SOS3*, and *NHX1* genes and modulation of stomatal behavior and gas exchange through enhanced K^+^ content are especially noteworthy. While demonstrating positive effects, NaHS exhibited a comparatively lesser impact than MT. The complex relationship between MT and H_2_S is revealed by inhibitory tests with PAG and *p*-CPA, highlighting MT’s role in reducing salinity, it operates through diverse pathways, including one involving H_2_S. These results bring up new possibilities for the prospective agricultural uses of MT and NaHS by shedding light on how they strengthen wheat plants’ resistance to salt stress.

## Data availability statement

The original contributions presented in the study are included in the article/supplementary material. Further inquires can be directed to the corresponding author.

## Author contributions

SK: Investigation, Methodology, Writing – original draft. AA: Writing – original draft. MF: Formal analysis, Writing – original draft. AA-H: Funding acquisition, Writing – original draft. AS: Validation, Writing – original draft. NK: Conceptualization, Supervision, Writing – review & editing.
