# Supplementary material for: Relative effects of melatonin and hydrogen sulfide treatments in mitigating salt damage in wheat
Source: Front Plant Sci. 2024 Jul 25;15:1406092. doi: 10.3389/fpls.2024.1406092 (PMC11306083; doi:10.3389/fpls.2024.1406092)
Supplement: Supplementary file 1 [file Table_1.docx]

Relative effects of melatonin and hydrogen sulfide treatments in mitigating salt damage in wheat

Sheen Khan^1^, Ameena Fatima Alvi^1^, Mehar Fatma^1^, Abdulrahman Al-Hashimi^2^, Adriano Sofo^3*^, Nafees A. Khan^1*^

^1^ Plant Physiology and Biochemistry Laboratory, Department of Botany, Aligarh Muslim University, Aligarh 202002, India

^2^ Department of Botany and Microbiology, College of Science, King Saud University, Riyadh 11451, Saudia Arabia

^3^Department of European and Mediterranean Cultures, Architecture, Environment, Cultural Heritage (DiCEM), University of Basilicata, 75100, Matera, Italy

*** Correspondence:**Corresponding Author
[adiano.sofo@unibas.it](mailto:adiano.sofo@unibas.it), [naf9.amu@gmail.com](mailto:naf9.amu@gmail.com)

# Supplementary Data

**Material and Methods**

**Determination of H_2_O_2_ Content**

500 mg of fresh leaf tissue was homogenized in ice-cold 200 mM perchloric acid (HClO_4_) and subsequently centrifuged at 1200 x g for 10 minutes. The resulting supernatant was neutralized with 4 M KOH. Further clarification involved centrifugation at 500× g for 3 minutes to eliminate insoluble potassium perchlorate. To assess H_2_O_2_ levels, the reaction mixture (1.5 mL) comprised 1 mL of the eluate, 80 µL of 3-methyl-2-benzothiazoline hydrazone, 400 µL of 12.5 mM 3-(dimethylamino) benzoic acid in 0.375 M phosphate buffer (pH 6.5), and 20 µL of peroxidase (0.25 unit). The reaction was initiated with the addition of peroxidase at 25 °C, and the resultant increase in absorbance was measured at 590 nm using a spectrophotometer.

**Determination of TBARS Content**

Fresh leaf samples (500 mg) were crushed in a solution comprising 0.25% 2-thiobarbituric acid (TBA) in 10% trichloroacetic acid (TCA) using a mortar and pestle. The mixture was then heated at 95 °C for 30 minutes, rapidly cooled in an ice bath, and subjected to centrifugation at 10,000× g for 10 minutes. To 1 mL of the resulting supernatant, 4.0 mL of 20% TCA containing 5% TBA was added. The absorbance of the supernatant was measured at 532 nm and adjusted for non-specific turbidity by subtracting the absorbance at 600 nm. The calculation of TBARS content was performed using an extinction coefficient of 155 mM^−1^ cm^−1^.

**Determination of Antioxidant Enzymes Activity**

**Superoxide Dismutase (SOD)**

Briefly, 5.0 mL of reaction mixture containing 5 mM HEPES (pH 7.6), 0.1 mM EDTA, 50 mM Na_2_CO_3_ (pH 10.0), 13 mM methionine, 0.025% (v/v) Triton X-100, 63 µmol NBT, and 1.3 µmol riboflavin was mixed with the enzyme-containing extract. The reactants were then placed in bright light (360 µmol m^−2^ s ^−1^ ) for 15 min, while a corresponding control was not illuminated to allow for the correction of background absorbance. One unit of SOD is defined as the amount of enzyme needed to inhibit NBT reduction by 50% (as measured by the absorbance at 560 nm).

**Ascorbate Peroxidase (APX)**

The assay mixture (1.0 mL) contained phosphate buffer (50 mM, pH 7.0), 0.1 mM EDTA, 0.5 mM ascorbate, 0.1 mM H_2_O_2_, and enzyme extract, and was observed at 290 nm for 1 min using a spectrophotometer. A decrease in absorbance was observed as soon as the reaction was started (i.e., upon the addition of H_2_O_2_). An extinction coefficient of 2.8 mM^−1^ cm^−1^ was used when computing APX activity. One unit of APX is defined as the amount necessary to decompose one µmol of substrate per min at 25 °C.

**Glutathione Reductase (GR)**

The reaction mixture (3.0 mL) contained phosphate buffer (25 mM, pH 7.8), 0.5 mM GSSG, 0.2 mM NADPH, and the enzyme extract. The reaction was initiated upon the addition of GSSG, and a decreasing trend in absorbance was immediately evident. An extinction coefficient of 6.2 mM^−1^ cm^−1^ was used when quantifying GR activity. One unit of enzyme is defined as the amount necessary to decompose one µmol of NADPH per min at 25 ^◦^C.

**GSH Content**

Reduced glutathione was assayed by an enzyme recycling procedure in which it was sequentially oxidized by 5, 5-dithiobis-2-nitrobenzoic acid (DTNB) and reduced by NADPH in the presence of GR. For specific assay of GSSG, GSH was masked by derivatization with 2-vinylpyridine. Fresh leaf tissues (500 mg) were ground in liquid nitrogen using mortar and pestle and suspended in 2 ml of 5% (w:v) sulfosalicylic acid. The centrifugation was done at 12,000×g for 10 min. A 300 μL aliquot of the supernatant was removed and neutralized by the addition of 18 μL 7.5 M triethanolamine. To determine concentrations of GSH plus GSSG, one 150 μL sample was then used. Another sample was pre-treated with 3 μL 2-vinylpyridine for 60 min at 20ºC to mask the GSH by derivatization, to allow the subsequent determination of GSSG alone. In each case, 50 μL aliquots of the two types of samples were mixed with 700 μL 0.3 mM NADPH, 100 μL DTNB, and 150 μl buffer containing 125 mM sodium phosphate, 6.3 mM EDTA (pH 6.5). A10 μL aliquot of GR (5 U ml^-1^) was then added and the change in absorbance at 412 nm was monitored at 30ºC. The standard curve was prepared from GSH, covering a range of 5-55 nmol. For oxidized glutathione, a standard curve covering a range of 1-5 nmol was used.

**Measurement of melatonin content**

To quantify melatonin (MT), High-Performance Liquid Chromatography (HPLC) was employed. Initially, the samples were homogenized with chloroform at 4 °C in the dark. Subsequently, the chloroform phase was purified using a Solid Phase Extraction (SPE) C18 cartridge after centrifugation at 4000×g for 5 minutes. For HPLC analysis, the particles from the evaporated extracts were reconstituted in methanol, using an HPLC system equipped with a 5 mm Hypersil ODS column and a fluorescence detector. The mobile phase consisted of methanol flowing at a rate of 1.0 mL min^−1^, and 20 mL of the sample was injected into the system. MT detection was achieved with an excitation wavelength set to 280 nm and an emission wavelength set to 348 nm.

**Measurement of H_2_S content**

To estimate leaf hydrogen sulfide (H_2_S) content, the formation of methylene blue from dimethyl-p-phenylenediamine in HCl was employed, following the procedure outlined by Xie et al. in 2014 with minor modifications. Fresh leaf samples (700 mg) were homogenized in 2.5 mL of Tris-HCl buffer (20 mM L^−1^, pH 6.8) containing 10 mM L^−1^ ethylenediaminetetraacetic acid (EDTA). The homogenate underwent centrifugation for 15 minutes at 4 °C and 12,000× g. For H_2_S trapping, 0.2 mL of 1% (w/v) zinc acetate was added to the supernatant (0.75 mL). After 30 minutes of development, 0.1 mL of 30 mM L^−1^ ferric chloride in 1.2 mol L^−1^ of HCl and 0.1 mL of 20 mM L^−1^ dimethyl-p-phenylenediamine dissolved in 7.2 mol L^−1^ of HCl were introduced. The formation of methylene blue was determined at 670 nm through spectrophotometric analysis. Different concentrations of NaHS were utilized as a standard curve, expressed as nmol g^−1^ fresh weight (FW).

**Determination of calvin cycle enzyme activity**

Rubisco activity was assessed using the method outlined by Usuda in 1985. Leaf tissue weighing 1.0 g underwent homogenization in a chilled mortar and pestle using an ice-cold extraction buffer containing 0.25 M Tris-HCl (pH 7.8), 0.0025 mM EDTA, 0.05 mM MgCl2, and 37.5 mg DTT for enzyme extraction. The resulting homogenate was then subjected to centrifugation at 10,000× g for 10 minutes at 4 °C. The supernatant obtained post-centrifugation was utilized for the enzyme assay. The reaction mixture comprised 100 mM Tris-HCl (pH 8.0), 10 mM MgCl2, 40 mM NaHCO3, 4.0 mM ATP, 0.2 mM NADH, 5.0 mM DTT, 0.2 mM EDTA, 1.0 U of glyceraldehyde-3-phosphate dehydrogenase, 1.0 U of 3-phosphoglycerate kinase, and 0.2 mM ribulose-1,5-bisphosphate (RuBP).

To estimate FBPase activity, the method outlined by Zhang et al. in 2014 was employed. Leaf samples were homogenized in 4 mL of buffer pre-cooled with ice, containing 50 mM MgCl_2_, 2 mM EDTA, 2% PVP, and 1% mercaptoethanol. The homogenates underwent centrifugation at 15,000×g for 20 minutes at 4 °C, and the resulting supernatant was used for determining the enzyme activity. The assay medium for FBP included 2–4 units per mL of phosphoglucoisomerase and glucose-6-phosphate dehydrogenase, along with 30 mM Hepes–KOH (pH 8.2), 5 mM MgCl_2_, 5 mM DTT, 0.5 mM NADP, and 5 mM FBP. The initiation of the assay involved adding FBP to the reaction, and the rate was measured 10 to 15 minutes after the commencement of the assay.

Spectrophotometric estimation was conducted for SBPase using method of Harrison et al., 1997. Leaf samples were finely ground in liquid nitrogen and then transferred to 1 mL of extraction buffer containing 50 mM Hepes (pH 8.2), 5 mM MgCl2, 1 mM EDTA, 1 mM EGTA, 10% glycerol, 2 mM benzamidine, 2 mM amino caproic acid, 0.5 mM PMSF, and 10 mM DTT. Following centrifugation, the supernatant was collected and passed through a NAP-10 column that had been pre-equilibrated with a desalting buffer. For the assay, 80 mL of assay buffer (50 mM Tris; pH 8.2, 15 mM MgCl2, 1.5 mM EDTA, 10 mM DTT, and 2 mM SBP) was added to 20 mL of protein samples, and the mixture was incubated at 25 °C for 5 minutes. The reaction was halted by adding 50 µL of 1 M perchloric acid. Subsequently, the samples were centrifuged for 5 minutes, and the supernatant was assessed for phosphate. A total of 850 µL of molybdate solution (0.3% ammonium molybdate in 0.55 M H2SO4) was incubated with 50 µL of samples and phosphate standards (0–0.5 mM NaH2PO4) for 10 minutes at room temperature. Following the addition of 150 µL of malachite green (0.035% malachite green, 0.35% polyvinyl alcohol), the samples underwent an additional 45-minute incubation at room temperature. The absorbance of the reaction mixture was then recorded at 620 nm using a spectrophotometer.

**Determination of Total Soluble Sugars, Total Non-Structural Carbohydrate (TSC) and Starch Content**

The procedure outlined by Xu et al. 2015 was employed for the quantification of soluble sugars. Top leaves that had fully expanded were collected for each treatment to estimate the soluble sugar content. After oven-drying at 80 °C, the leaf samples were finely ground into powder. Using 10 mL of 80% ethanol, the dried sample (100 mg) underwent extraction and was heated in a water bath at 80–85 °C for 30 minutes. Following centrifugation of the extract, the supernatant was transferred to a 100 mL volumetric flask, and this process was repeated three times. The alcohol extract was evaporated in a water bath at 80–85 °C. The flask was then filled with a mixture of all three supernatants, and 100 mL of distilled water was added. Anthrone reagent was employed to assess the amount of soluble sugars in the extract, and a spectrophotometer measured the absorbance of the reaction mixture at 630 nm. Soluble sugars and starch content are presented as mg g^−1^ dry weight.

Starch estimation was determined spectrophotometrically at 620 nm using anthrone reagent with glucose as the standard, as outlined in protocol of Kuai et al., 2014. Dried leaf tissues were ground and filtered through a 1 mm sieve. Approximately 100 mg of the powdered material was added to 5 mL of 80% ethanol in a 10 mL centrifuge tube. The mixture was initially incubated for 30 minutes at 80 °C in a water bath shaker and then centrifuged for 5 minutes at 4000×g. Subsequently, the pellets were extracted with 80% ethanol, and the ethanol was removed through evaporation. The starch in the residue was released after 15 minutes in a boiling bath with 2 mL of distilled water, followed by cooling to room temperature. Next, 9.2 mol L^−1^ HClO_4_ (2 mL) was employed to hydrolyze leaf starch for 15 minutes. After the addition of distilled water (4 mL), the samples were centrifuged at 4000×g for 10 minutes. A second extraction of the residue was performed using 4.6 mol L−1 HClO_4_ (2 mL). The supernatants were collected, combined, and diluted with distilled water until reaching a final volume of 25 mL.

The NSC content was the sum of the soluble sugar content and starch content.

**RNA extraction and Quantitative RT-PCR**

The formula used for the calculation of relative gene expression was 2^− ΔΔCT^ method (Livak and Schmittgen, 2001). The sequence of primer pairs used for quantitative RT-PCR:

**Table S1**. Primer pairs used for quantitative RT-PCR.

| **S.No.** | **Gene** | | | **Forward primer** | | **Reverse primer** |
| --- | --- | --- | --- | --- | --- | --- |
| 1 | *SOS1* | | | CATCCGTATCTGGGCTGCCA | | TACAGCAGCGCCAAGCAAAA |
| 2 | *SOS2* | | | GGCTTTCGCGAATCGCATCA | | GCGACCAACCTCGTACTTGC |
| 3 | *SOS3* | | | GAGTCCGACCTGTGCCTCTC | | CCTGAGTGATGCCGGGTTCT |
| 4 | *NHX1* | | | GTGCCTCGGCCTGACAGTAT | | AGACGGGTCGCATGAAGGAG |
| **Reference gene primer sequences used for quantitative RT-PCR** | | | | | | |
| 1 | | *Actin* | TGGACTCTGGTGATGGTGTTA | | CAATGAGGGATGGCTGGAAAA | |

**References**

Okuda, T., Matsuda, Y., Yamanaka, A., and Sagisaka, S. (1991). Abrupt increase in the level of hydrogen peroxide in leaves of winter wheat is caused by cold treatment. *Plant Physiol.* 97, 1265-1267.

Dhindsa, R. S., Plumb-Dhindsa, P. A. M. E. L. A., and Thorpe, T. A. (1981). Leaf senescence: correlated with increased levels of membrane permeability and lipid peroxidation, and decreased levels of superoxide dismutase and catalase. *J. Exp. Bot.* 32, 93-101.

Beyer Jr, W. F., and Fridovich, I. (1987). Assaying for superoxide dismutase activity: some large consequences of minor changes in conditions. *Anal. Biochem.* 161, 559-566.

Giannopolitis, C. N., and Ries, S. K. (1977). Superoxide dismutases: I. Occurrence in higher plants. *Plant Physiol.* 59, 309-314.

Nakano, Y., and Asada, K. (1981). Hydrogen peroxide is scavenged by ascorbate-specific peroxidase in spinach chloroplasts. *Plant Cell Physiol.* 22, 867-880.

Foyer, C. H., and Halliwell, B. (1976). The presence of glutathione and glutathione reductase in chloroplasts: a proposed role in ascorbic acid metabolism. *Planta*, 133, 21-25.

Anderson, M.E. (1985). Determination of glutathione and glutathione disulfide in biological samples. Methods in Enzymology, Academic Press, 113, 548–554.

Sehar, Z., Fatma, M., Khan, S., Mir, I. R., Abdi, G., and Khan, N. A. (2023). Melatonin influences methyl jasmonate-induced protection of photosynthetic activity in wheat plants against heat stress by regulating ethylene-synthesis genes and antioxidant metabolism. *Sci, Rep.*  13, 7468.

Xie, Y., Zhang, C., Lai, D., Sun, Y., Samma, M. K., Zhang, J., and Shen, W. (2014). Hydrogen sulfide delays GA-triggered programmed cell death in wheat aleurone layers by the modulation of glutathione homeostasis and heme oxygenase-1 expression. *J. Plant Physiol.* 171, 53-62.

Xu, W., Cui, K., Xu, A., Nie, L., Huang, J., and Peng, S. (2015). Drought stress condition increases root to shoot ratio via alteration of carbohydrate partitioning and enzymatic activity in rice seedlings. *Acta Physiol.Plant.* 37, 1-11.

Kuai, J., Liu, Z., Wang, Y., Meng, Y., Chen, B., Zhao, W., and Oosterhuis, D. M. (2014). Waterlogging during flowering and boll forming stages affects sucrose metabolism in the leaves subtending the cotton boll and its relationship with boll weight. *Plant Sci.* 223, 79-98.

Usuda, H. (1985). The activation state of ribulose 1, 5-bisphosphate carboxylase in maize leaves in dark and light. *Plant Cell Physiol.* 26, 1455-1463.

Zhang, K., Liu, H., Tao, P., and Chen, H. (2014). Comparative proteomic analyses provide new insights into low phosphorus stress responses in maize leaves. *PLoS One* 9, e98215.

Harrison, E. P., Willingham, N. M., Lloyd, J. C., and Raines, C. A. (1997). Reduced sedoheptulose-1, 7-bisphosphatase levels in transgenic tobacco lead to decreased photosynthetic capacity and altered carbohydrate accumulation. *Planta* 204, 27-36.

Gautam, H., Fatma, M., Sehar, Z., Iqbal, N., Albaqami, M., and Khan, N. A. (2022). Exogenously-sourced ethylene positively modulates photosynthesis, carbohydrate metabolism, and antioxidant defense to enhance heat tolerance in rice. *Int. J. Mol. Sci.* 23, 1031.

Livak, K. J., and Schmittgen, T. D. (2001). Analysis of relative gene expression data using real-time quantitative PCR and the 2^− ΔΔCT^ method. *Methods* 25, 402-408.

**
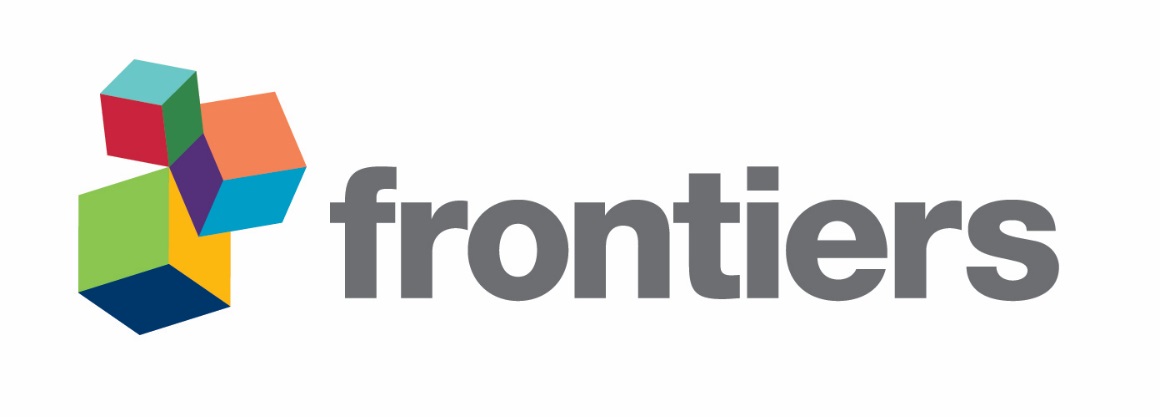
**
